# Correlated STORM-homoFRET imaging reveals highly heterogeneous membrane receptor structures

**DOI:** 10.1016/j.jbc.2022.102448

**Published:** 2022-09-05

**Authors:** Amine Driouchi, Scott D. Gray-Owen, Christopher M. Yip

**Affiliations:** 1 Department of Biochemistry, 1 King's College Circle, University of Toronto, Toronto, Ontario, Canada; 2 Institute of Biomedical Engineering, University of Toronto, Toronto, Ontario, Canada; 3 Terrence Donnelly Centre for Cellular & Biomolecular Research, University of Toronto, Toronto, Ontario, Canada; 4 Department of Molecular Genetics, University of Toronto, 1 King’s College Circle, Toronto, Ontario, Canada; 5 Department of Chemical Engineering & Applied Chemistry, University of Toronto, Toronto, Ontario, Canada

**Keywords:** super-resolution microscopy, STORM, homoFRET, membrane proteins, oligomers, nanobodies, clustering, CEACAM1, carcinoembryonic antigen-related cellular adhesion molecule 1, FRET, Förster resonance energy transfer, m-Venus, monomeric Venus, td-Venus, tandemly expressed dimeric Venus, PSF, point spread functions, SMLM, single molecule localization microscopy, STORM, stochastic optical reconstruction microscopy, TIRF, total internal reflection fluorescence

## Abstract

Mapping the self-organization and spatial distribution of membrane proteins is key to understanding their function. Developing methods that can provide insight into correlations between membrane protein colocalization and interactions is challenging. We report here on a correlated stochastic optical reconstruction microscopy/homoFRET imaging approach for resolving the nanoscale distribution and oligomeric state of membrane proteins. Using live cell homoFRET imaging of carcinoembryonic antigen-related cellular adhesion molecule 1, a cell-surface receptor known to exist in a complex equilibrium between monomer and dimer/oligomer states, we revealed highly heterogeneous diffraction-limited structures on the surface of HeLa cells. Furthermore, correlated super-resolved stochastic optical reconstruction microscopy imaging showed that these structures comprised a complex mixture and spatial distribution of self-associated carcinoembryonic antigen-related cellular adhesion molecule 1 molecules. In conclusion, this correlated approach provides a compelling strategy for addressing challenging questions about the interplay between membrane protein concentration, distribution, interaction, clustering, and function.

Identifying the factors that govern the spatial-temporal distribution and interaction of membrane proteins is critical for addressing key questions in cell biology. These include determining the structural underpinnings of intercellular engagement and the mechanisms of effector signaling upon receptor activation. Identifying the key structure-function relationships relies upon quantifying protein dynamics and association state (1, 2, 3, 4). Techniques such as Förster resonance energy transfer (FRET) (5, 6, 7) and fluorescence correlation spectroscopy (8, 9) are often employed to quantify and spatially map protein–protein interactions, clustering, self-association, and dynamics. One commonly used approach for characterizing self-association is homoFRET, which occurs due to energy transfer between identical fluorophores that can act as either donors or acceptors. In homoFRET, nonradiative energy transfer between identical fluorophores located within the requisite Förster distance results in depolarized emission upon excitation with linearly polarized light. As such, this method reports on interactions that occur over distances less than 10 nm and with transfer efficiencies dependent on the fluorophore, local structure, and extent of conformational flexibility (5, 6, 10). The result of homoFRET imaging is a diffraction-limited pixel-wise anisotropy map that reports the spatial distribution of self-association.

Although FRET-based approaches provide insights into the proximity of two interacting fluorophores, the diffraction limit classically limits the extent of spatial localization. Developments in super-resolution fluorescence microscopy or single molecule localization microscopy (SMLM) are allowing researchers to resolve objects with a localization precision of 20 to 100 nm, often revealing subdiffraction limit structural features (11, 12). These techniques can be subdivided into two categories: ensemble imaging approaches, which include stimulated emission depletion (1, 13, 14, 15, 16) and structured illumination microscopies (16, 17, 18, 19), and SMLM, which includes stochastic optical reconstruction microscopy (STORM) (20, 21, 22) and photoactivated localization microscopy (23). A key limitation for SMLM lies in determining whether the localized structures are in fact interacting or are simply clustered in a noninteracting fashion. This is further complicated by the very nature of the SMLM acquisition and image processing algorithms, which typically rely on stochastic excitation of, and emission from, individual fluorophores. This challenge prompted us to develop an integrated approach that would marry the high spatial resolution of SMLM with self-association data provided by homoFRET. Since homoFRET images are diffraction-limited, the pixel-wise *r*-values can only report on the average association state within the pixel, and not the true spatial distribution. In principle, correlating super-resolution and homoFRET images would provide new insights into the relationships between oligomeric state and spatial distribution (24). Here we describe the methodology behind a correlative STORM/homoFRET technique (Figs. 1 and S1). When applied to membrane or membrane-associated proteins, this correlated approach can reveal their nanoscale distribution and oligomerization or self-association state.

**Figure 1:**
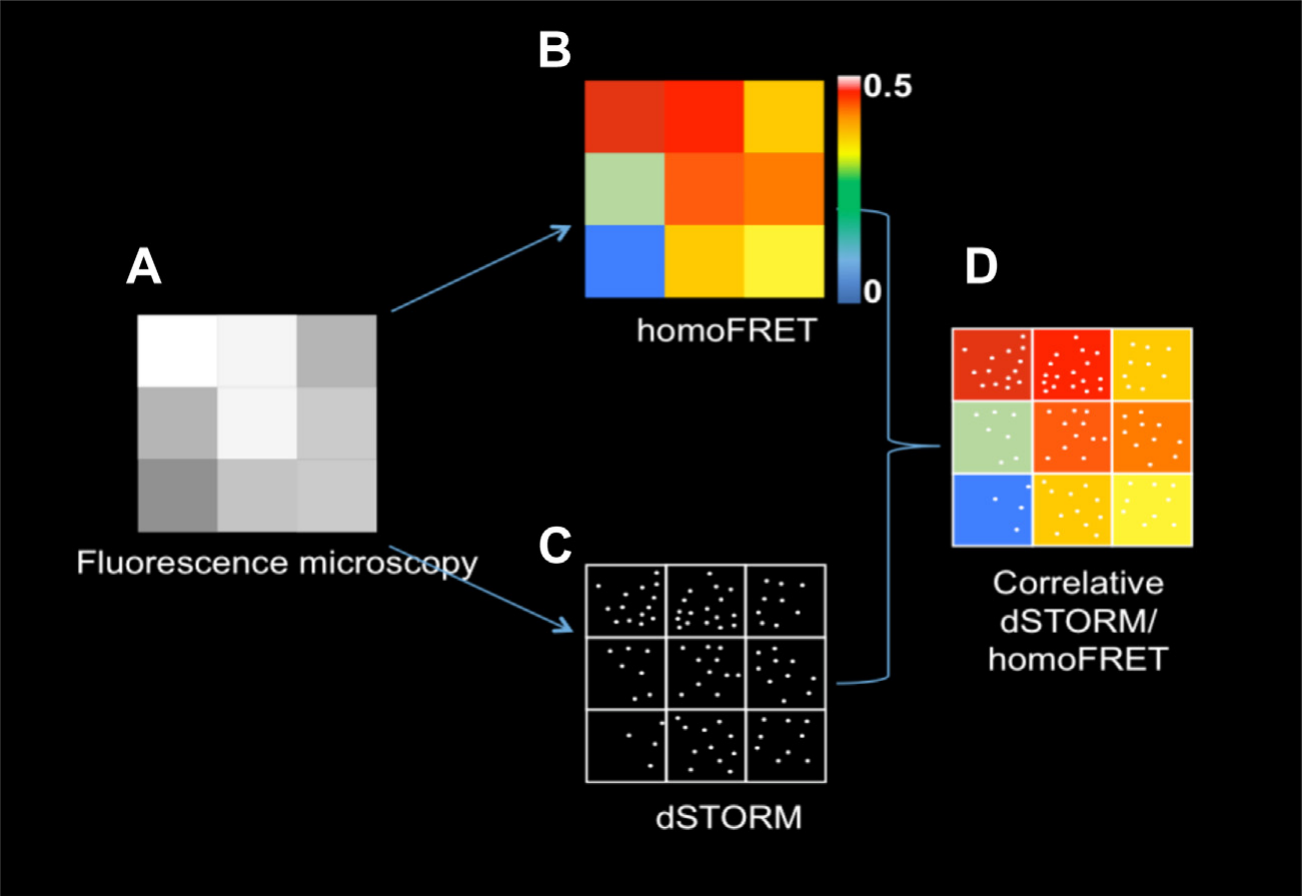
**Schematic representation of the correlated STORM/homoFRET approach using a model 3 x 3 pixel image.***A*, diffraction-limited fluorescence microscopy with each pixel displaying a *gray-scale* intensity value. *B*, in a homoFRET image, each pixel is assigned an *r*-value representing the pixel’s average anisotropy, with lower values reflecting increased self-association. *C*, an idealized STORM image showing individual localizations without their respective localization uncertainty. *D*, correlated STORM/homoFRET allows matching of the true spatial distribution with the pixel-wise anisotropy values. FRET, Förster resonance energy transfer; STORM, stochastic optical reconstruction microscopy.

### CEACAM1-4L, a transmembrane cell-adhesion molecule

For the purposes of the present work, we focused on the prototype member of the carcinoembryonic antigen-related cellular adhesion molecule (CEACAM) family, which are cell surface glycoproteins involved in homophilic and heterophilic intercellular interactions involved in cellular growth, differentiation, tumorigenesis, inflammation, and infection (25). Despite CEACAMs’ importance in health and disease, the nature of CEACAM interactions and their mechanisms of signaling remain poorly understood. The full-length splice variant of CEACAM1 has an extracellular domain consisting of an amino-terminal IgV-like domain and three IgC2-like domains, with all these domains being heavily glycosylated (26, 27). This protein is anchored into the membrane *via* a transmembrane sequence and a 76-amino acid cytoplasmic domain that contains two immunoreceptor tyrosine-based inhibitory motifs, although some variants lack these sequences (25). While it has been known that CEACAM1 can exist as a monomer, a dimer, or higher-order oligomer (Fig. 2) and that different oligomeric forms engage different downstream signaling cascades, how these complexes are distributed remains unknown (28, 29, 30). Furthermore, the dynamics and interchange between these oligomeric states remain poorly characterized. These questions prompted us to develop a correlative STORM/homoFRET-based approach for characterizing the nanoscale distribution of CEACAM1-4L monomers and oligomers on the cell surface. In this study, homoFRET measurements were performed using YFP-labeled CEACAM1-4L, while STORM imaging was accomplished through a YFP-specific camelid single domain antibody labeled with Alexa Fluor 647 (AF647).

**Figure 2:**
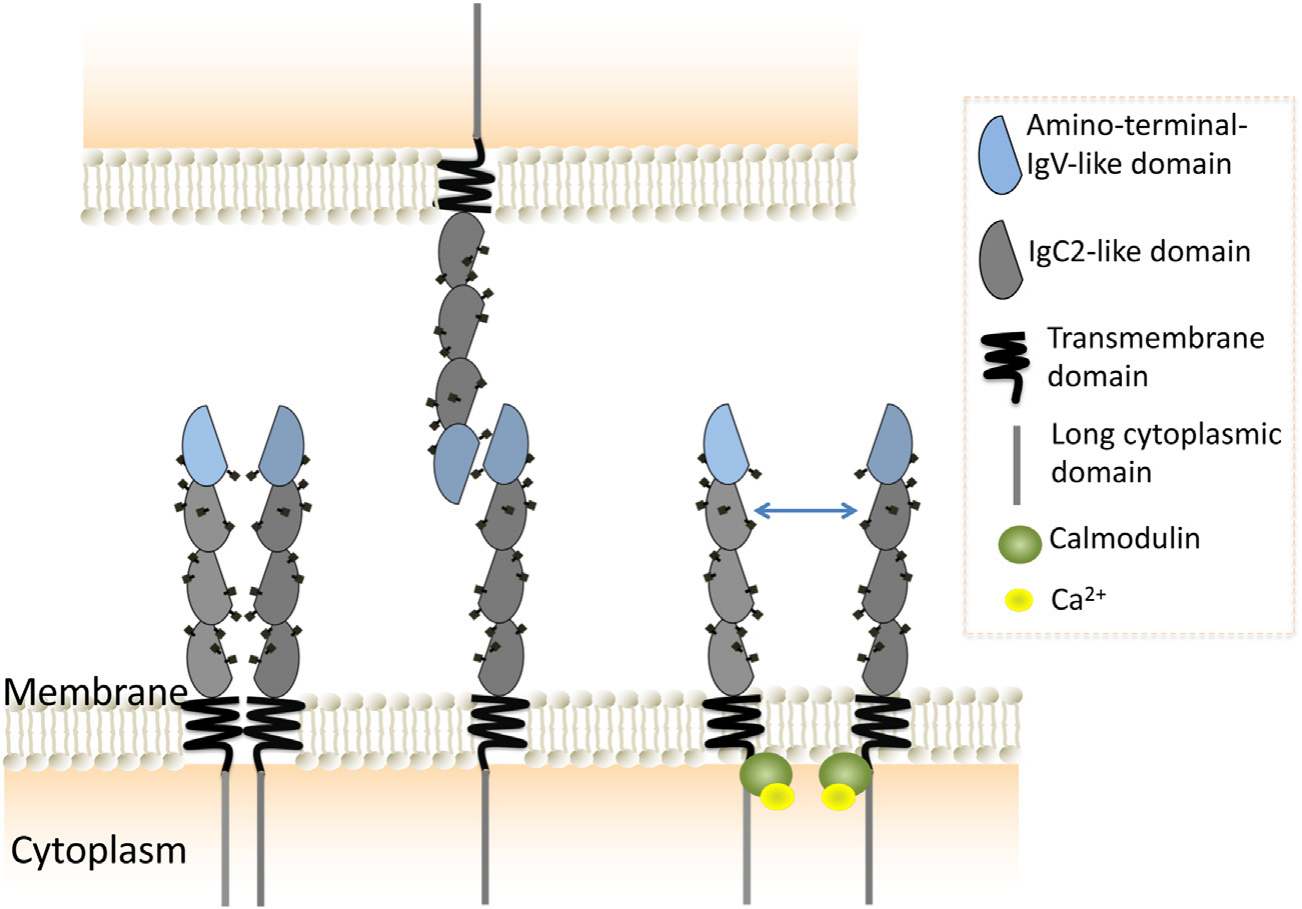
**CEACAM1-4L is known to exist as a monomer, dimer, or higher-order oligomer.** This equilibrium is regulated by key cofactors, including disruption by binding of calcium-loaded calmodulin to the cytoplasmic domain. CEACAM1, carcinoembryonic antigen-related cellular adhesion molecule 1.

## Results

### Steady-state fluorescence anisotropy of CEACAM1-eYFP

The human endocervically derived HeLa cells are one of the few epithelial cell lines that lacks endogenous CEACAMs, allowing ectopic expression of individual CEACAM1-4L family members to ascribe function. Past work has shown that ectopically expressed CEACAM1-4L (CEACAM1) can transition between the monomeric and cis-dimeric forms seen in cell lines that naturally express this protein (28, 31). Thus, for these studies, we have used HeLa cells transiently transfected with a chimeric allele encoding CEACAM1 with a carboxyl terminus fused eYFP (CEACAM1-eYFP).

To establish the range of values attainable, total internal reflection fluorescence (TIRF)-based homoFRET measurements were determined using HeLa cells expressing soluble monomeric Venus (m-Venus) and the tandemly expressed dimeric Venus (td-Venus) (Fig. S4). Note that, due to the soluble nature of both m-Venus and td-Venus, we expected anisotropy values noticeably lower to what would be expected for a more restricted membrane-bound target. Before this and all subsequent experiments, the G-factor correction factor was measured to correct for potentially varying light sensitivities of the detectors that capture parallel and perpendicular polarizations. In addition, we have shown that the resulting pixel-wise anisotropy map is independent of the given intensity values and is, therefore, expressed as a ratio between the two captured emission channels (Fig. S5).

**Figure 3:**
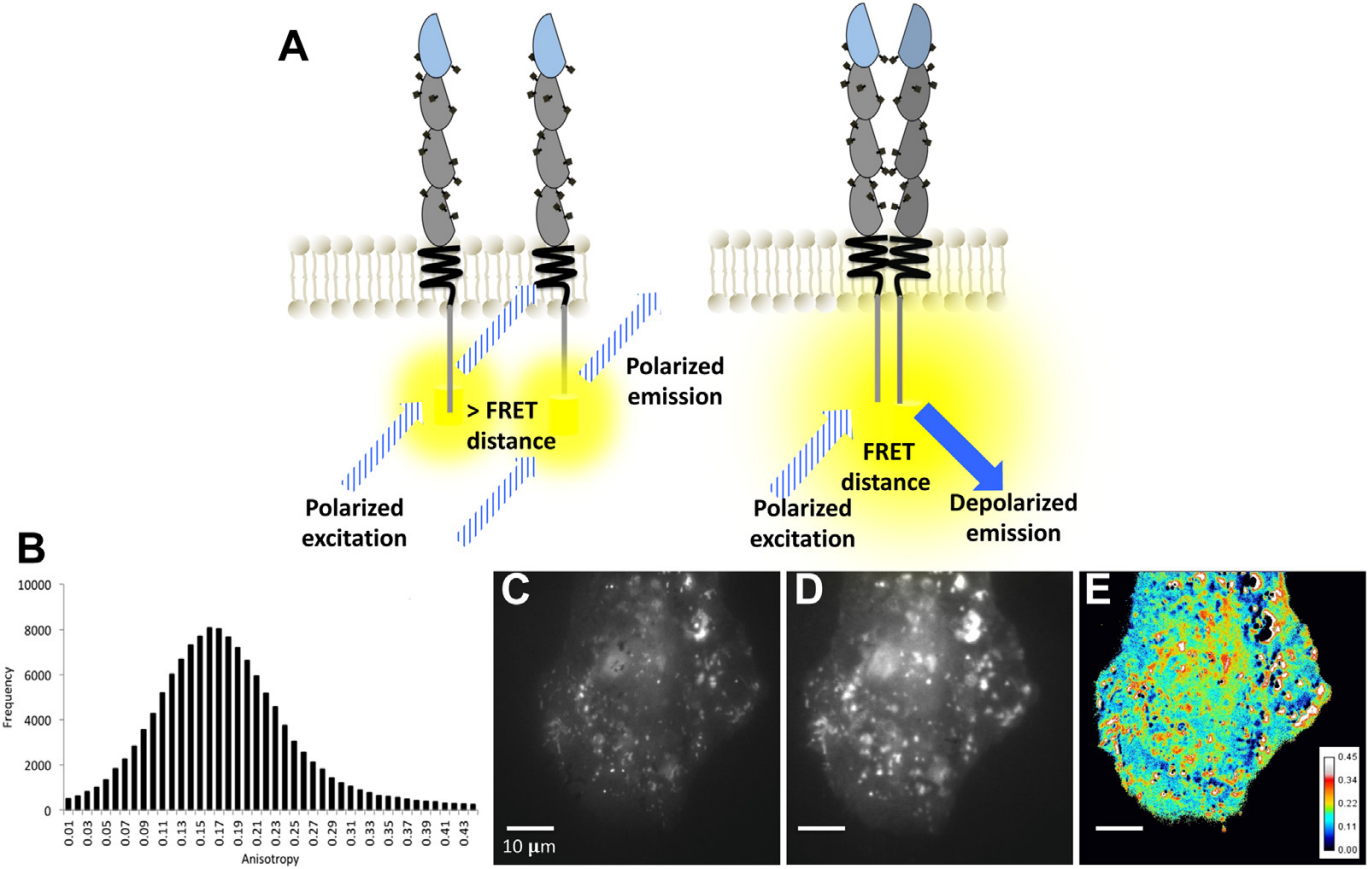
**HomoFRET provides an anisotropy map representative of the distribution of monomers and oligomers.***A*, schematic showing depolarized emission as a result of FRET when two identical fluorophores are within the requisite FRET distance. *B*, distribution of anisotropies within HeLa cell transiently transfected with CEACAM1-eYFP. *C*, polarized (F_ll_) diffraction-limited image. *D*, depolarized (F_⊥_) diffraction-limited image. *E*, anisotropy map illustrating the heterogeneous distribution of CEACAM1 monomers and oligomers, corresponding to high (*red*) and low anisotropy values (*blue*), respectively. Scale bar for panels (*C–E*) denotes 10 μm. CEACAM1, carcinoembryonic antigen-related cellular adhesion molecule 1; FRET, Förster resonance energy transfer.

Live-cell TIRF-homoFRET imaging of CEACAM1-eYFP in HeLa cells revealed the presence of both diffuse and irregularly bright regions. Counterintuitively, the low intensity/diffuse regions were comprised of predominantly dimeric or oligomeric CEACAM1 with anisotropy values ranging from 0.1 to 0.2, while the high intensity/clustered regions appeared more monomeric, with anisotropy values ranging from 0.3 to 0.45 (Fig. 3). Saturated pixels are not taken into consideration in the homoFRET calculation as the anisotropy calculation requires two intensity values from the collected images, while pixels with low gray-scale values (close to background levels) are removed using a threshold value kept constant for all analyzed cells (Fig. 3*E*). The presence of cell-to-cell variability suggests that the observed equilibrium is regulated by the cell, stages of the cell cycle, interactions with neighboring cells, and overall CEACAM1-specific biology (Fig. S2). In addition, we performed homoFRET experiments on a well-characterized CEACAM1 mutant known to be unable to form cis-dimers. These experiments revealed a dramatic shift to higher anisotropy values for this monomeric mutant (Fig. S6). We subsequently performed homoFRET experiments on a CEACAM1 mutant (R43S/Q44L) incapable of forming trans-homophilic interactions (28). In this case, the histogram distribution is shifted toward lower pixel values, indicative of enrichment in cis-dimers and oligomers (Fig. S6). Although not the focus of this paper, these insights highlight the fact that most oligomeric CEACAM1 exists likely in the cis (nonmonomeric) conformation. Overall, these experiments highlight the utility of this approach for studying self-association states.

There is clearly a nonuniform spatial distribution of anisotropies across the surface of each cell, with an unexpected inverse correlation between CEACAM1 density (reflected by eYFP intensity) and the proportion of CEACAM1 that is in an oligomeric state (reflected by lower anisotropy values). However, the diffraction-limited spatial resolution of homo-FRET prevents clear characterization of CEACAM1 organization within these regions. To overcome this limitation, we applied STORM imaging to the same samples that had been characterized by TIRF-homoFRET imaging.

### Single-domain antibody labeling for STORM

**Figure 4:**
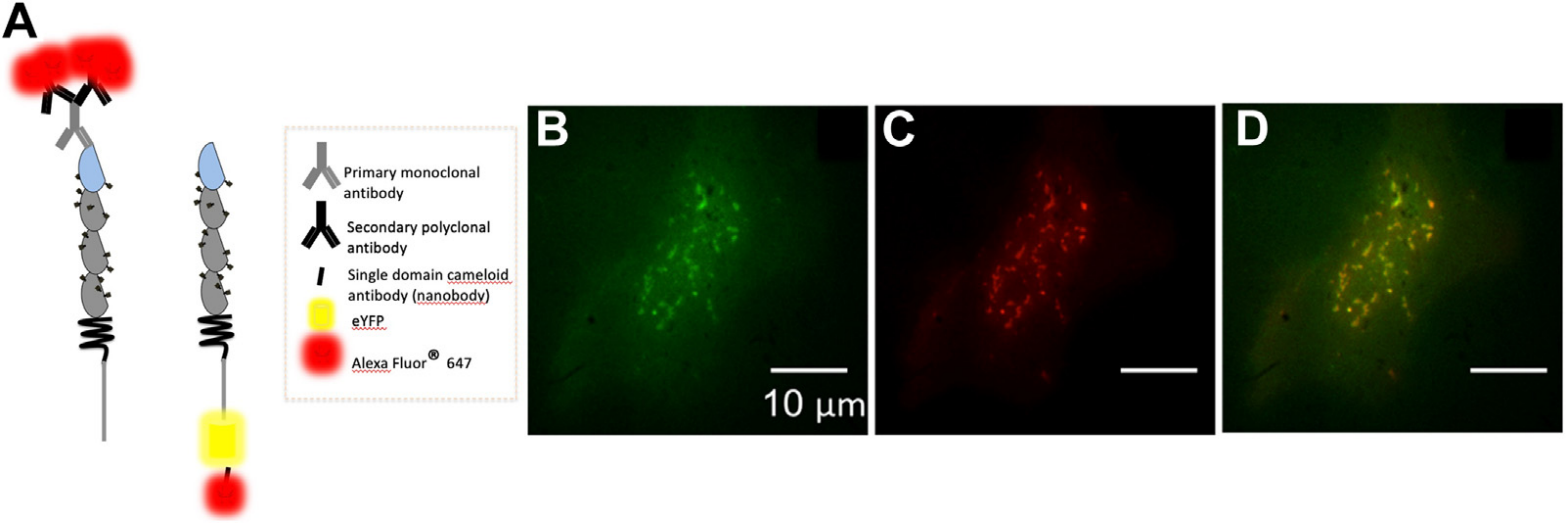
**Using an eYFP-specific nanobody facilitates correlative STORM/homoFRET due to its low number of fluorophores and their close proximity to eYFP**. *A*, schematic illustration to highlight spatial differences between CEACAM1 labeling using a monoclonal antibody targeting the extracellular domain with an AF647-labeled secondary polyclonal antibodies *versus* directly conjugated single domain camelid antibody (nanobody). Each primary antibody can bind several polyclonal antibodies, and each secondary antibody typically bears four to eight fluorophores, whereas the camelid antibodies possess one or two. CEACAM1 labeling using an eYFP-specific nanobody. Representative fluorescence image of (*B*) CEACAM1-eYFP, (*C*) CEACAM1-nanobody-AF647, (*D*) merged eYFP, and AF647 image. CEACAM1, carcinoembryonic antigen-related cellular adhesion molecule 1; FRET, Förster resonance energy transfer; STORM, stochastic optical reconstruction microscopy.

For STORM imaging, CEACAM1-eYFP is labeled with an AF647-labeled eYFP-specific camelid single domain antibody (Fig. 4) (32, 33). A key challenge in the use of antibodies for STORM imaging lies in the size and flexibility of the antibodies themselves, which are typically ∼13 nm long; this can result in significant variability in the localization. To address this, we elected to use a nanobody comprised solely of the 12 to 15 kDa variable domain of a camelid antibody heavy chain. This nanobody is ∼1.5 to 2.5 nm in size and has been specifically engineered to bind to the β-barrel of GFP and its derivatives (including eYFP) with high specificity and affinity (34). Moreover, nanobodies only bear 1 to 2 fluorophores, which both reduces the likelihood of overlocalizing the individual antibody (or nanobody) during STORM imaging and minimizes the generation of self-clustering artifacts. Overlapping point spread functions (PSFs) can lead to mislocalization and underestimation or overestimation of single emitters. This can lead to the artifactual creation of clusters or nanodomains, even in the case of a homogeneous distribution. As described by Sauer *et al.* (35), in order to minimize false multiple-fluorophore localizations, the lifetime of the off-state has to be significantly longer than that of the on-state. In this way, the distances between individual blinks are large enough to minimize PSF overlaps. Here, we aim to measure the average distances between individual PSFs for both the antibody and nanobody labeling strategies to confirm that we have achieved sufficient separation.

We randomly selected 10 frames from the 1000 frames dataset acquired for a given cell and super-resolved the individual frames prior to conducting a nearest-neighbor analysis on the resulting coordinates (Fig. S8). To compare and contrast the antibody and nanobody labeling approaches, three replicate experiments were conducted to account for sample preparation variability. The average nearest neighbor distances for PSFs for each of the antibody (n = 4307 PSFs) and nanobody (n = 5231 PSFs) labeling strategies were 1725 nm (±19) and 2040 nm (±26), respectively (*p* < 0.005 using a two-sample *t* test). These results strongly indicate that both strategies generate raw datasets with comparable localization densities that should maximize the signal to noise ratio (and hence the localization precision), while minimizing instances of mislocalization and self-clustering artifacts. These results highlight the appropriateness of our labeling strategy for STORM imaging of protein nanoclusters.

For STORM imaging, the CEACAM1-eYFP HeLa cells were fixed with paraformaldehyde, permeabilized, and then labeled with 1 nM of AF647-conjugated anti-YFP nanobody for 30 min (Fig. S7). Analysis of conventional wide-field fluorescence images (Fig. 4, *B–D*) revealed that the eYFP- and AF647-labeled regions were highly colocalized (Pearson coefficient = 0.861 ± 0.05 measured from six cells for each of three replicate experiments (Fig. S3). In addition, since the AF647-labeled nanobody is bound to an eYFP which itself is bound to a flexible linker attached to the cytoplasmic domain of CEACAM1, we characterized the small but non-negligible fluctuation of the fluorophore’s position over a 1 min acquisition period (Fig. S9). We found this fluctuation to be well below the localization precision of STORM imaging. These data suggest that this approach provides for highly specific high-affinity labeling and thus enables correlative STORM/homoFRET experiments.

### Quantifying the impact of cell permeabilization and single-domain antibody labeling on eYFP anisotropy

**Figure 5:**
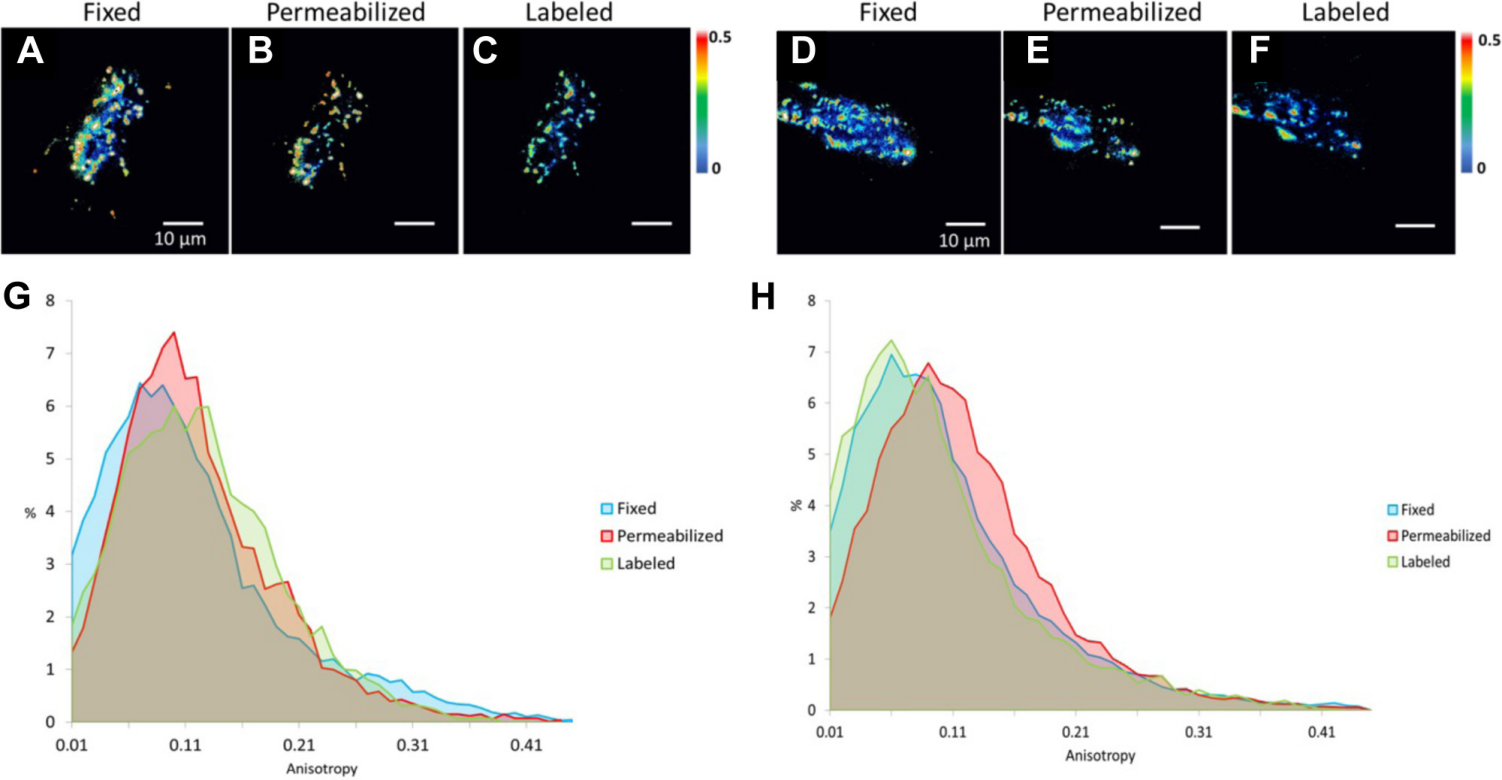
**Measuring potential distortions caused by the single domain antibody-dependent immunostaining**. Images of two representative cells depicting anisotropy measurements following cell fixation using 4% paraformaldehyde (*A* and *D*), cell permeabilization using 0.1% Triton X-100 (*B* and *E*), nanobody-labeling (*C* and *F*). Histograms (*G* and *H*) of the anisotropy distribution of the cells depicted in *A–C* and *D–F*, respectively, illustrating the overall pixel-wise change in oligomerization states upon disruption through fixation, permeabilization, and labeling. Note that the cell shown in (*A–C*) is the same cell shown in Figure 4, *B*–*D*, now depicted as pixel-wise anisotropy images for each of fixed, permeabilized and labeled conditions.

While homoFRET of CEACAM1-eYFP can be performed on live cells, staining with the anti-eYFP nanobody requires fixation and permeabilization of the cells. To assess how these steps may affect correlation of the anisotropy and STORM datasets, anisotropy measurements were performed after each fixation and permeabilization step (Fig. 5). While close inspection of the corresponding images indicates that the cluster distributions and morphologies remained largely unaffected by these steps (Fig. S10), permeabilization did produce an apparent shift toward lower anisotropies or oligomeric CEACAM1.

The cumulative anisotropy plots revealed no significant shift in anisotropy upon fixation, whereas permeabilization caused a small shift to the right (Fig. 5, *G* and *H*). However, after detergent removal during nanobody labeling, the cumulative anisotropy curve appeared to revert to values comparable to those originally obtained in the fixed but nonpermeabilized samples. This suggests that detergent-induced distortion occurring during permeabilization may be transient in nature due to the low concentration of Triton X-100 used. This is consistent with prior work showing that only at concentrations above 0.20 mM does Triton X-100 exerts an irreversible effect on membrane permeability (35). Overall, fixation, permeabilization, and labeling as performed in our experiments appear to minimally alter self-association states, clustering, and overall cell morphology (see Experimental procedures).

### Investigating the nanoscale organization of nanobody-labeled CEACAM1 using dSTORM

**Figure 6:**
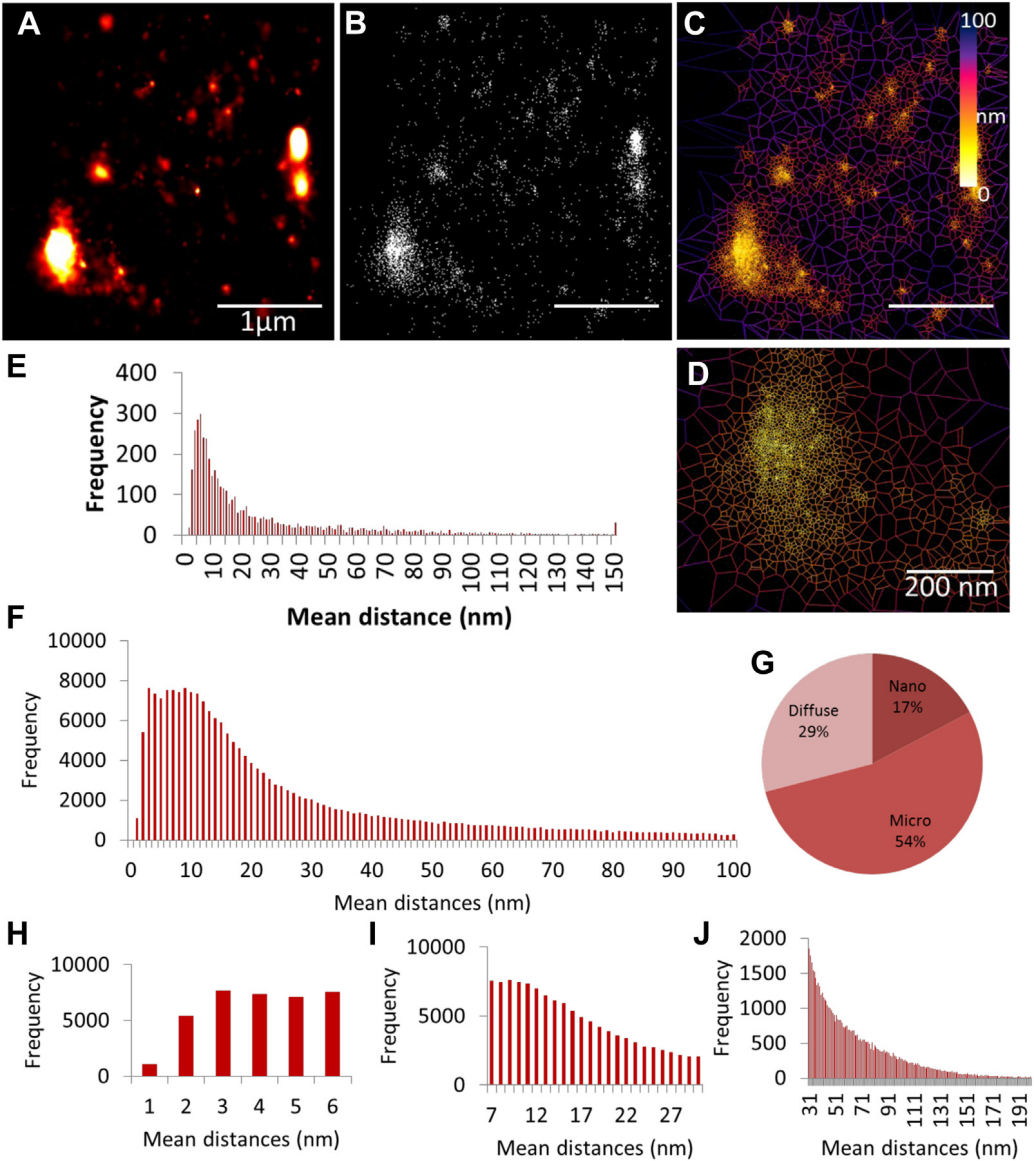
**Voronoi tessellation enables distinction between three different classes of clusters**. *A*, Gaussian rendering of super-resolved CEACAM1 on HeLa cell, representative of the localization uncertainty of each point. *B*, scatter plot of super resolved CEACAM1. The points are presented without the localization uncertainty. *C*, Voronoi tessellation. *D*, Voronoi tessellation of an enlarged CEACAM1 cluster (compare scale bars in *A* and *D*). *E*, distribution of mean distances for tessellated ROI. *F*, cumulative frequency plot of mean distances collected from four cells. *G*, pie chart showing the average clustering of CEACAM1 localizations. *H*, nanocluster (1–6 nm) frequency plot of mean distances. *I*, microcluster (7–30 nm) frequency plot of mean distances. *J*, diffuse (>31 nm) regions frequency plot of mean distances. CEACAM1, carcinoembryonic antigen-related cellular adhesion molecule 1.

Our diffraction-limited TIRF microscopy revealed that CEACAM1 is distributed either in a diffuse manner or as clusters exhibiting various sizes and shapes. To understand the nature of the CEACAM1 species within the clusters, Voronoi tessellation–based cluster analysis was performed on super-resolved CEACAM1 datasets using the SR-Tesseler package (Fig. 6). Voronoi tessellation subdivides a set of coordinates into polygonal regions by tracing the bisector to each of two nearest neighbors. For each coordinate, the mean distance to the adjacent nearest neighbors can be calculated, generating a mean distance distribution per cell or region of interest. Regions of interest can be thresholded out and clusters identified by setting the number of localizations and cluster area minima and maxima (Fig. S11). Remarkably, this clustering analysis revealed the presence of a consistent trimodal distribution of the mean distance histogram, suggestive of the presence of diffuse regions, microclusters, and nanoclusters (Fig. S12). Since the work is conducted in fixed cells, the apparent differences in nanoclustering frequency across analyzed cells likely reflects the short-lived nature of nanoclusters. However, we have captured live-cell confocal datasets that have clearly shown the persistence and presence of microclusters in both the basolateral and apical membrane of transiently transfected HeLa cells (Fig. S17). Here, microclusters and nanoclusters are defined as a function of cluster density that appears to correlate well with cluster area and diameter. Similar higher density regions have been reported for other membrane proteins and are thought to exist in order to restrict their activity to intercellular contact points or to co-localize with subcellular structures (36, 37, 38, 39). While not core to this methods paper, Fig. S15 presents results highlighting a preferential enrichment in monomers within intercellular contact sites.

Once the different cluster groups were identified, we measured area, density, circularity, and diameter for each of the nanocluster and microclustered regions from four cells. While circularities are similar (0.55–0.60), the average cluster diameter for microclusters and nanoclusters were 215 nm ± 27 and 49 nm ± 28, respectively (Fig. S18, *B* and *C*). Finally, nanoclusters appear almost 3 times denser than microclusters (Fig. S17*D*). These differences may reflect functional differences between the microclusters and nanoclusters and differences in their association to the lipid membrane and cytoskeletal components.

### Correlating homoFRET and dSTORM

**Figure 7:**
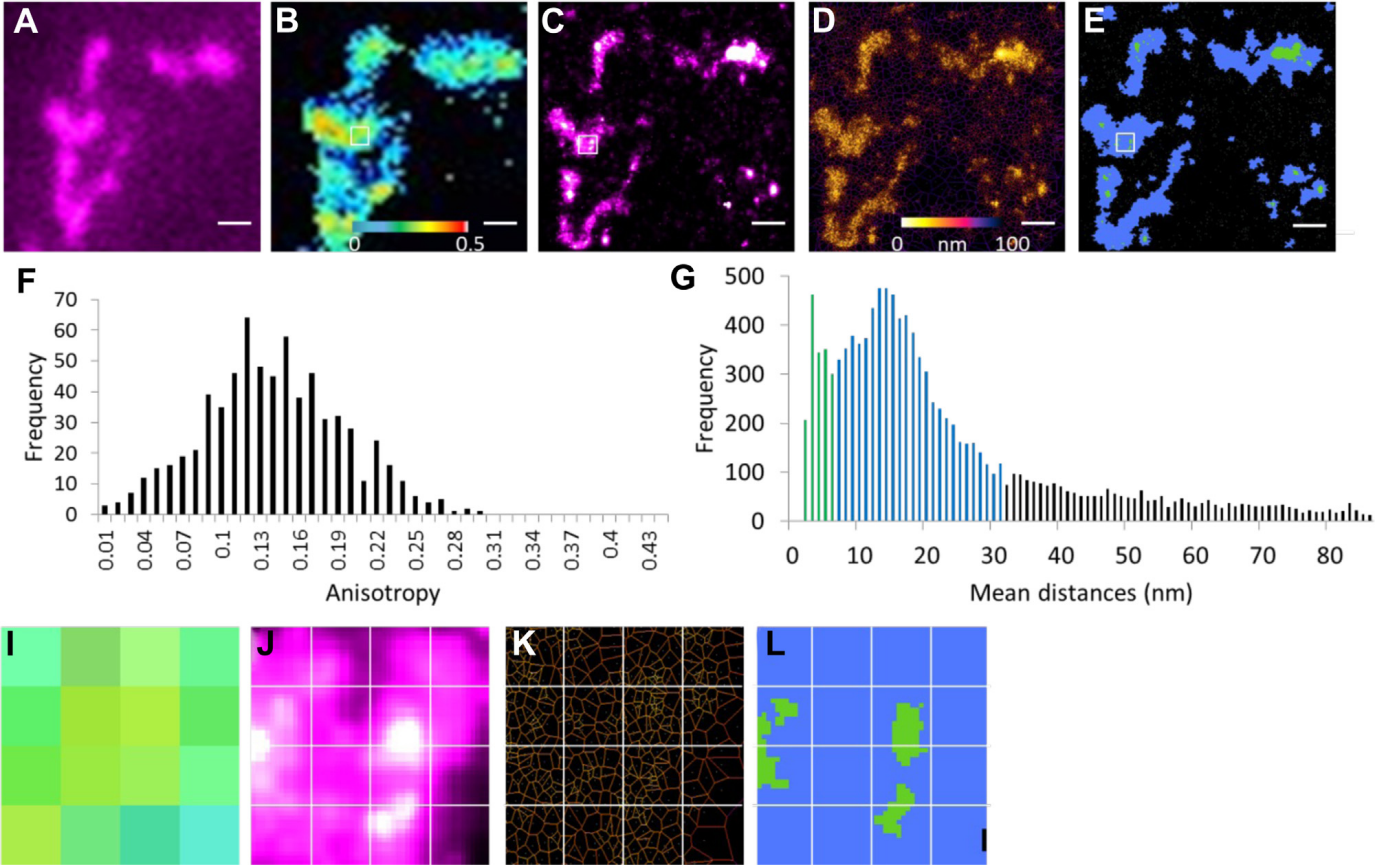
**Correlating STORM/homoFRET enables to uncover the preferential spatial distribution of CEACAM1 monomers and oligomers**. *A*, diffraction-limited image of CEACAM1 labeled with anti-eYFP AF-647-conjugated nanobody. *B*, homoFRET anisotropy map of CEACAM1-eYFP. *C*, Super-resolved image of CEACAM1 labeled with AF-647-conjugated anti-eYFP nanobodies. *D*, Voronoi tessellation segmentation. *E*, segmentation into microclustered and nanoclustered areas. *F*, histogram of anisotropy values for CEACAM1-eYFP cluster in panel *B*. *G*, mean distances distribution derived from Voronoi tessellation segmentation [*green—*nanoclusters; *blue*—microclusters; *black*—nonclustered diffuse regions]. Panels *I*–*L*, 4 × 4 pixels with a pixel size of 127 nm scaled up to reveal detail within regions denoted by *white boxes* in panels *B*, *C*, and *E*, respectively. (*I*) anisotropy map, (*J*) super-resolved CEACAM1-AF647, (*K*) Voronoi tessellation segmentation; (*L*) Segmentation into microclustered and nanoclustered areas. Scale bar for panels *A–E* denotes 1 μm. CEACAM1, carcinoembryonic antigen-related cellular adhesion molecule 1; FRET, Förster resonance energy transfer; STORM, stochastic optical reconstruction microscopy.

After cluster analysis *via* Voronoi tessellation, the diffraction-limited homoFRET image was scaled up without interpolation to a 6502 × 6502 pixel 16 bit image and then overlaid on the corresponding STORM image. CEACAM1 clusters of different shapes, sizes, and density can be correlated to their respective per-pixel *r* values and, conversely, pixels with a given anisotropy can be assigned with a subdiffraction pixel nanodistribution (Fig. S13). While we cannot completely discount the possibility of processing artifacts in the registration of the super-resolution STORM with the homoFRET datasets, the fact that the individual point-spread functions do span several adjacent pixels suggests that this effect is likely minimal (Fig. 7).

What becomes clear from this analysis is that pixels with equal anisotropy values display a wide range of possible nanoscale distributions (Fig. 7, *I–L*). It is notable that low anisotropy pixels tended to be devoid of nanoclustered regions, instead appearing to be comprised of microclustered and diffuse CEACAM1. These findings suggest that CEACAM1 organizes preferentially into three characteristic states: a diffuse distribution of oligomers, microclusters of oligomers and monomers, and nanoclusters of monomers (Fig. S14). We note that similar cluster classifications were reported by others studying T-cells using SMLM (40). In addition to this approach, we have also correlated the anisotropy map to its corresponding nearest neighbor map (Fig. S16), which revealed that the higher anisotropy pixels at the core of the cluster have, on average, localizations that are closer to their nearest neighbor. This is consistent with our observation of a negative correlation between self-association and protein density.

## Discussion

The present work has demonstrated that combining STORM with homoFRET imaging enables direct determination of the spatial and oligomeric state distributions of surface membrane proteins. In the case of CEACAM1, we have shown that this glycoprotein can exist in three distinct clustered states, each with its own unique self-association characteristics: diffuse regions consisting of oligomers present on the flatter cell surface, microclusters of oligomers and monomers, and nanoclusters of monomers that tend to be within cell protrusions. These arrangements are not resolvable using conventional diffraction-limited approaches. The unexpected inverse relationship between CEACAM1 density and its propensity to be in an oligomeric state provides new insight into the dynamics of this important receptor.

The transmembrane domain of CEACAM1-4L causes it to be preferentially enriched within cell membrane regions that are dense in lipoproteins, cholesterol, and GM1 gangliosides, which are presumed to reflect more ordered lipid microdomains (41). Given that the cytoplasmic domain of CEACAM1 is thought to be phosphorylated by Src family kinases within these lipid domains, it is enticing to consider that either this phosphorylation and/or the phosphotyrosine-dependent docking of downstream effector proteins maintains monomeric CEACAM1 within high-density regions. While actin is not required for CEACAM1-mediated bacterial uptake (41), its cytoplasmic domain can interact with the actin at sites of cell–cell contact (42), implying different states of anchorage to the cytoskeleton also occur. Notable in this regard, we have captured results suggesting that actin and ezrin may colocalize CEACAM1-4L clusters within actin corrals within high-intensity actin regions (unpublished results). Future work must consider whether the distribution and oligomeric state of CEACAM1 determines its association with lipid and/or cytoskeletal components or vice versa.

**Figure 8:**
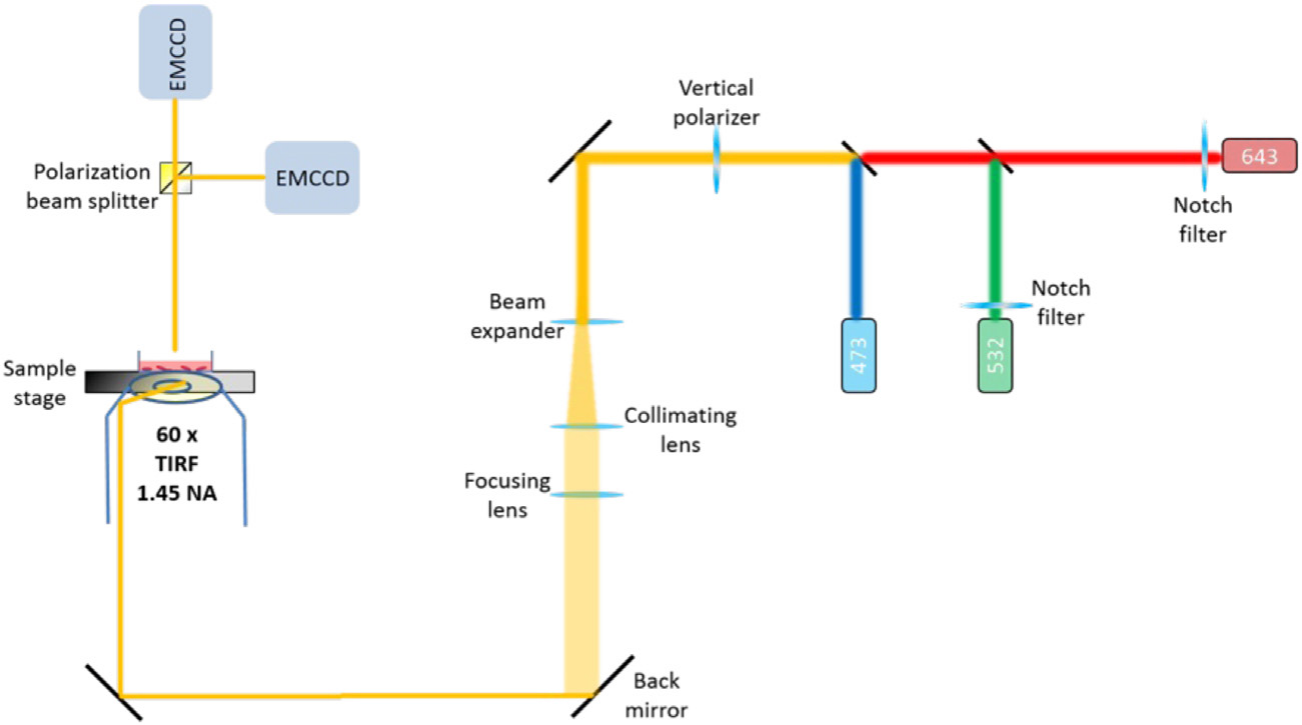
**Schematic of the microscope used to conduct STORM/homoFRET experiments**. FRET, Förster resonance energy transfer; STORM, stochastic optical reconstruction microscopy.

While our studies focused on the full-length variant of CEACAM1, the relative ease of implementation, both in terms of microscope configuration (Fig. 8) and labeling strategy makes this approach applicable to virtually all membrane proteins. This method can be generalized to other fluorescent proteins. The availability of other anti-xFP-specific nanobodies conjugated to other photoswitchable organic fluorophores that span the visible spectrum, as well as other fluorescent xFP-mutants opens the door for multicolor STORM/homoFRET experiments, simultaneously monitoring the arrangement of multiple cellular proteins. We have also highlighted the importance of selecting a suitable cluster analysis strategy in order to categorize single molecule localizations into suitable groups. In our hands, Voronoi tessellation proved to be the most relevant approach as it allows for the subdivision of points into polygonal regions without user bias. Nonetheless, although these determinations were performed without considering localization uncertainty, the observed differences in density are still representative of differences in labeling density that reflects the distribution of CEACAM1. The resulting mean distance or density distributions revealed a trimodal distribution for CEACAM1 in the analyzed cells, showing three discrete populations that were indistinguishable under diffraction-limited fluorescence microscopy (Fig. S12). Although similar classes of clusters were observed by others in studies of T-cells using SMLM techniques (37), it must be recognized that factors such as PSF overlap, the effect of multiple labels, and the selection of appropriate reconstruction and cluster analysis do play a role in cluster characterizations. Additional work is underway to characterize the dynamics of the putative cluster distributions that we have seen in the present work. This will further aid in clarifying the nature of CEACAM1 self-association and the role of clustering on its function. In addition, we are currently investigating the validity of our correlated approach to the intercellular interface. Having established a system capable of revealing the subtle, nanoscale 2D arrangement of CEACAM1 at the membrane, future studies can now aim to understand how different CEACAM1 isoforms and mutants compare and how they are affected by intercellular interaction among CEACAM family members, providing new insights regarding their respective roles and the evolutionary need for such a large array of glycoproteins.

## Experimental procedures

### Cell culture and transfection

HeLa cells were cultured in 75 cm^2^ Falcon culture flasks with supplemented RPMI Medium 1640 (1×) + L-glutamine (Gibco by Life Technologies) containing 10% fetal bovine serum (Gibco by Life Technologies), 1% glutamax, and 0.05% penicillin/streptomycin. Cells were incubated at 37 °C with 5% CO_2._ Upon reaching 70 to 80% confluence, cells were detached using 1× trypsin:PBS for 5 min and were seeded on Nunc Lab-Tek II 8-well chamber slides (Thermo Scientific) with RPMI containing fetal bovine serum, glutamax, and penicillin/streptomycin. Cells were left to adhere and divide for 24 to 36 h prior to transfection. Transfection with the CEACAM1-eYFP-encoding was conducted by resuspending 10 μl of the plasmid in 87 μl of RPMI-free serum for 5 min, followed by the addition of 3 μl polyethylenimine and incubation at room temperature (22 °C). Twenty-five microliter of the total 100 μl transfection solution is added to each imaging chamber. Transfected cells are incubated for 16 to 18 h, upon which media are switched to transfection reagent-free media.

### Microscope setup

TIRF microscopy was performed on a home-built TIRF microscopy system integrated with an Olympus FluoView 500 confocal microscope using an IX-70 base (Olympus Canada) using a high numerical aperture 60× oil-immersion objective (NA = 1.45, Olympus Japan). A thin layer of index-matching oil (n = 1.518) was used to optically couple the objective to the glass surface of the Nunc Lab-Tek II eight well chamber slides (Thermo Scientific). Excitation of eYFP was achieved using an analog modulated 473 nm diode laser (DHOM-L-150 mW, Suzhou Daheng Optics & Fine Mechanics Co, Ltd). Excitation of AF647 was achieved using an analog modulated 643 nm laser (Power Technology, Model LDCU5/A109) with a maximum measured power of 90 mW at the source. A clean-up notch filter (ZET642/20×, Chroma) was used to spectrally clean the excitation. A polarization beam splitter was used to separate between polarized and depolarized emission (PBS251, Thorlabs) (Fig. 8). Fluorescent images are captured using two water-cooled eXcelon-equipped Evolve 512 EMCCD camera (Photometrics) using μ-Manager (version 1.4.19). Under these conditions, we measure an effective pixel size of 127 nm. Imaging for a single fixed cell was performed over a 30 min period, and 5 to 10 cells were imaged. Note that our microscope did not possess a hardware-based perfect focus in place to account for z-drift during the acquisition period.

### TIRF homoFRET

TIRF-based homoFRET images are collected as pairs of images where F_ll_ is the polarized fluorescence intensity collected through a polarizer oriented parallel to the excitation and F_⊥_ representing the depolarized fluorescence intensity collected through a polarizer oriented perpendicular to the excitation source. All images were collected using 500 ms exposure period, gain of 1, and an electron multiplier gain of 100. To correct for detector sensitivity for both F_ll_ and F_⊥_ that arise from differences in their transmissivities through the various optical components of the microscope, an experimental G factor is determined. Once the G factor is determined, anisotropy (*r*) can be determined through the following equation:

$$\text{r}=\frac{{\text{I}}_{∥}-G{\text{I}}_{⊥}}{{\text{I}}_{∥}+2G{\text{I}}_{⊥}}$$

The G factor value of 0.93 was determined by calculating the intensity ratio of I_ll_/I_⊥_ for a 100 nM fluorescein standard solution. G factor measurements are performed whenever an optical component is modified or moved into the optical train of the microscope.

### Preparation of labeled nanobodies

Nanobodies were purchased from Chromotek as GFP-Trap. GFP-Trap nanobodies were conjugated to AF647 using conventional conjugation protocols. For labeling, 50 μl of the 1 mg/ml protein solution was dialyzed into 0.2 M NaHCO_3_, pH 8.2 using a mini-dialysis unit (Pierce, molecular weight cutoff = 3500 Da). Succinimidyl-ester of AF647 was dissolved at 10 mg/ml in DMSO, which was added in excess to the nanobodies. The solution was incubated for 1 h at room temperature. Excess dye was removed *via* buffer exchange into PBS using a desalting column. Conjugated nanobodies were stored at 4 °C.

### Antibody and nanobody labeling

HeLa cells at 70% confluence were fixed in 4% formaldehyde in phosphate buffered saline (PBS) for 10 min. Cells were washed with PBS and permeabilized with 0.1% Tx-100 for 30 min. Cells were thoroughly washed with PBS to remove Tx-100 residues and were then blocked with 10% goat serum in PBS for 1 h. The primary CEACAM1 antibody (D14HD11) was incubated in goat serum for 2 h prior to washing with PBS. The secondary antibody (AF-647 conjugated) was incubated for 2 h in PBS. Cells were finally washed with PBS to remove excess antibody. For nanobody labeling, cells were fixed and permeabilized similarly to the protocol used for antibody labeling. The nanobody was then directly added into the cells and allowed to incubate briefly prior to STORM imaging.

### Colocalization of eYFP to nanobody-AF647

Colocalization analysis was conducted using the JACoP (Just Another Colocalization Plugin) ImageJ plugin. Analysis was conducted on six cells from three independent experiments. Costes automatic thresholding was used prior to calculating the Pearson coefficient between eYFP and AF647 images (43). Arithmetic mean and standard error of the mean were calculated and shows the high degree of colocalization between eYFP and AF647 channels, indicative of the large fraction of eYFPs bearing a nanobody.

### STORM imaging

In order to initiate stochastic photoswitching for STORM, photoswitching buffer is added prior to imaging. The buffer consists of 50 mM cysteamine (2-mercaptoethylamine, Sigma-Aldrich), 40 μg/ml catalase (from bovine liver, aqueous solution, Sigma-Aldrich), 0.5 mg/ml glucose oxidase (*Aspergillus niger*, Sigma-Aldrich), and 50% w/v glucose (D-glucose, Sigma-Aldrich) diluted in pH 7.4 PBS buffer. This buffer provides conditions that yield a high photon count for AF647 and modulates the photophysical properties of AF647 by scavenging oxygen and creating a reducing environment. A 643 nm laser is set to a power of 20 mW, as measured after the objective, which is used to drive AF647 to an off-state prior to having subsets of fluorophores coming back on in a stochastic manner over the acquisition period. To reconstruct a super-resolved image, 10,000 to 15,000 images were acquired, each with an exposure time of 30 ms. This number of frames was chosen to satisfy the condition of the Nyquist-Shannon sampling theory, which requires single localization separation distance to be half of the localization precision in order to be able to claim a resolution equal to the average localization precision of approximately 20 to 25 nm (43).

### STORM processing

Images are processed using ThunderSTORM (version 1.3) and the maximum likelihood localization algorithm localization parameter. Following localization and reconstruction, the coordinates of single emitters are filtered based on their localization precision (uncertainty value) and photon count in order to discard electronic noise. Specifically, uncertainty histograms are generated for each cell and experiment. We found that thermal and electronic noise resulted in uncertainties of [0–6] nm, while sample noise affected most localizations with a localization precision above 40 nm. Localizations with a neighbor within ∼1 nm are merged into a single localization in order to correct for self-clustering artifacts arising from multiple blinks of the same fluorophore or dual labels on a nanobody. The ThunderSTORM cross-correlation function was used to account for drift. Despite the care taken in accurately preprocessing and postprocessing blinks and due to the stochastic nature of photoswitching, the variation in the number of fluorophores per protein (albeit small), and overall imaging conditions, the super-resolved localization coordinates do not offer an absolute measurement (*i.e.*, the ability to count the number of proteins in a region) of protein count but a rather a statistically robust, relative quantitative representation of protein distribution and clustering.

### STORM cluster analysis

Several clustering algorithms have been used by other groups on sets of coordinates obtained from SMLM techniques with a preference for Ripley’s K function, pair-correlation analysis and density-based spatial clustering analysis with noise (40, 44). While useful, these methods present some limitations as they require user-assigned analysis parameters, which will skew the resulting clustering analysis (44, 45, 46). Here, we use Voronoi tessellation, which is a geometric method that subdivides space into polygonal areas (47). The region covered by all the polygons forms a mosaic of tiles of varying sizes that define what is called as a Voronoi diagram. In this approach, each polygon is centered around one localization, hence describing single molecules neighborhoods with their respective properties. Since a single STORM reconstructed image would contain 10^5^ to 10^6^ localizations depending on the sample, SR-Tesseler uses a sweep line algorithm to generate a Voronoi diagram in a matter of seconds (33 s for a full 512 × 512 pixels field of view with approximately 400,000 localizations). We use the SR-Tesseler software for this purpose. Note that localization uncertainty is not weighted into the cluster analysis.

### Nearest neighbor analysis to characterize the median distance between nearest neighbor localizations

Each localization is assigned a nearest neighbor defined as the closest localization in the super-resolved dataset. Localization uncertainties are not weighted in the distance calculation (code available in supplementary information).

## Data availability

All raw data are available upon request to: christopher.yip@utoronto.ca

## Supporting information

This article contains supporting information.

## Conflict of interest

The authors declare that they have no conflicts of interest with the contents of this article.

## References

[1] Kellner R.R., Baier C.J., Willig K.I., Hell S.W., Barrantes F.J. (2007). Nanoscale organization of nicotinic acetylcholine receptors revealed by stimulated emission depletion microscopy. Neuroscience.

[2] Heller J., Rusakov D. (2015). Subcellular distribution and trafficking of astroglial receptors monitored with super-resolution microscopy. Glia.

[3] Eggeling C. (2015). Studying plasma membrane bioactivity with super-resolution STED microscopy. FEBS J..

[4] Zhuang X. (2015). Illuminating biology at the nanoscale with super-resolution fluorescence microscopy. FEBS J..

[5] Piston D.W., Rizzo M.A. (2008). FRET by fluorescence polarization microscopy. Methods Cell Biol.

[6] Rizzo M.A., Piston D.W. (2005). High-contrast imaging of fluorescent protein FRET by fluorescence polarization microscopy. Biophys. J..

[7] Roy R., Hohng S., Ha T. (2008). A practical guide to single-molecule FRET. Nat. Met..

[8] Machan R., Wohland T. (2014). Recent applications of fluorescence correlation spectroscopy in live systems. FEBS Lett..

[9] Singh A.P., Wohland T. (2014). Applications of imaging fluorescence correlation spectroscopy. Curr. Opin. Chem. Biol..

[10] Cameron W.D., Bui C.V., Hutchinson A., Loppnau P., Gräslund S., Rocheleau J.V. (2016). Apollo-NADP(+): a spectrally tunable family of genetically encoded sensors for NADP(+). Nat. Met..

[11] Hell S.W., Wichmann J. (1994). Breaking the diffraction resolution limit by stimulated-emission - stimulated-emission-depletion fluorescence microscopy. Opt. Lett..

[12] Klar T.A., Engel E., Hell S.W. (2001). Breaking Abbe's diffraction resolution limit in fluorescence microscopy with stimulated emission depletion beams of various shapes. Phys. Rev. E Stat. Nonlin Soft Matter Phys..

[13] Klar T.A., Dyba M., Hell S.W. (2001). Stimulated emission depletion microscopy with an offset depleting beam. Appl. Phys. Lett..

[14] Westphal V., Blanca C.M., Dyba M., Kastrup L., Hell S.W. (2003). Laser-diode-stimulated emission depletion microscopy. Appl. Phys. Lett..

[15] Hein B., Willig K.I., Hell S.W. (2008). Stimulated emission depletion (STED) nanoscopy of a fluorescent protein-labeled organelle inside a living cell. Proc. Natl. Acad. Sci. U. S. A..

[16] Hell S.W., Sahl S.J., Bates M., Zhuang X., Heintzmann R., Booth M.J. (2015). The 2015 super-resolution microscopy roadmap. J. Phys. D-Applied Phys..

[17] Ball G., Demmerle J., Kaufmann R., Davis I., Dobbie I.M., Schermelleh L. (2015). SIMcheck: a toolbox for successful super-resolution structured illumination microscopy. Sci. Rep..

[18] Moerner W.E. (2015). Nobel Lecture: single-molecule spectroscopy, imaging, and photocontrol: foundations for super-resolution microscopy. Rev. Mod. Phys..

[19] Tang Y.Q., Dai L., Zhang X., Li J., Hendriks J., Fan X. (2015). SNSMIL, a real-time single molecule identification and localization algorithm for super-resolution fluorescence microscopy. Sci. Rep..

[20] Rust M.J., Bates M., Zhuang X. (2006). Sub-diffraction-limit imaging by stochastic optical reconstruction microscopy (STORM). Nat. Met..

[21] Benke A., Manley S. (2012). Live-cell dSTORM of cellular DNA based on direct DNA labeling. Chembiochem.

[22] Lehmann M., Gottschalk B., Puchkov D., Schmieder P., Schwagerus S., Hackenberger C.P. (2015). Multicolor caged dSTORM resolves the ultrastructure of synaptic vesicles in the brain. Angew. Chem. Int. Ed. Engl..

[23] Betzig E., Patterson G.H., Sougrat R., Lindwasser O.W., Olenych S., Bonifacino J.S. (2006). Imaging intracellular fluorescent proteins at nanometer resolution. Science.

[24] Oreopoulos J., Gray-Owen S.D., Yip C.M. (2017). High density or urban sprawl: what works best in biology?. ACS Nano.

[25] Gray-Owen S.D., Blumberg R.S. (2006). CEACAM1: contact-dependent control of immunity. Nat. Rev. Immunol..

[26] Nagaishi T., Pao L., Lin S.H., Iijima H., Kaser A., Qiao S.W. (2006). SHP1 phosphatase-dependent T cell inhibition by CEACAM1 adhesion molecule isoforms. Immunity.

[27] Chen C.J., Kirshner J., Sherman M.A., Hu W., Nguyen T., Shively J.E. (2007). Mutation analysis of the short cytoplasmic domain of the cell-cell adhesion molecule CEACAM1 identifies residues that orchestrate actin binding and lumen formation. J. Biol. Chem..

[28] Patel P.C., Lee H.S., Ming A.Y., Rath A., Deber C.M., Yip C.M. (2013). Inside-out signaling promotes dynamic changes in the carcinoembryonic antigen-related cellular adhesion molecule 1 (CEACAM1) oligomeric state to control its cell adhesion properties. J. Biol. Chem..

[29] Klaile E., Vorontsova O., Sigmundsson K., Müller M.M., Singer B.B., Ofverstedt L.G. (2009). The CEACAM1 N-terminal Ig domain mediates cis- and trans-binding and is essential for allosteric rearrangements of CEACAM1 microclusters. J. Cell Biol..

[30] Müller M.M., Klaile E., Vorontsova O., Singer B.B., Obrink B. (2009). Homophilic adhesion and CEACAM1-S regulate dimerization of CEACAM1-L and recruitment of SHP-2 and c-Src. J. Cell Biol..

[31] Hunter I., Sawa H., Edlund M., Obrink B. (1996). Evidence for regulated dimerization of cell-cell adhesion molecule (C-CAM) in epithelial cells. Biochem. J..

[32] Platonova E., Winterflood C.M., Junemann A., Albrecht D., Faix J., Ewers H. (2015). Single-molecule microscopy of molecules tagged with GFP or RFP derivatives in mammalian cells using nanobody binders. Methods.

[33] Dempsey G.T., Vaughan J.C., Chen K.H., Bates M., Zhuang X. (2011). Evaluation of fluorophores for optimal performance in localization-based super-resolution imaging. Nat. Met..

[34] Wachter R.M., Elsliger M.A., Kallio K., Hanson G.T., Remington S.J. (1998). Structural basis of spectral shifts in the yellow-emission variants of green fluorescent protein. Struct. Folding Des..

[35] Koley D., Bard A.J. (2010). Triton X-100 concentration effects on membrane permeability of a single HeLa (Koley and Bard 2010) cell by scanning electrochemical microscopy (SECM). Proc. Natl. Acad. Sci. U. S. A..

[36] Gasparrini F., Feest C., Bruckbauer A., Mattila P.K., Müller J., Nitschke L. (2016). Nanoscale organization and dynamics of the siglec CD22 cooperate with the cytoskeleton in restraining BCR signalling. EMBO J..

[37] Hu Y.S., Cang H., Lillemeier B.F. (2016). Superresolution imaging reveals nanometer- and micrometer-scale spatial distributions of T-cell receptors in lymph nodes. Proc. Natl. Acad. Sci. U. S. A..

[38] Gabor K.A., Stevens C.R., Pietraszewski M.J., Gould T.J., Shim J., Yoder J.A. (2013). Super resolution microscopy reveals that caveolin-1 is required for spatial organization of CRFB1 and subsequent antiviral signaling in zebrafish. PLoS One.

[39] Rossier O., Giannone G. (2016). The journey of integrins and partners in a complex interactions landscape studied by super-resolution microscopy and single protein tracking. Exp. Cell Res..

[40] Sengupta P., Jovanovic-Talisman T., Skoko D., Renz M., Veatch S.L., Lippincott-Schwartz J. (2011). Probing protein heterogeneity in the plasma membrane using PALM and pair correlation analysis. Nat. Met..

[41] Muenzner P., Bachmann V., Kuespert K., Hauck C.R. (2008). The CEACAM1 transmembrane domain, but not the cytoplasmic domain, directs internalization of human pathogens *via* membrane microdomains. Cell Microbiol..

[42] Sadekova S., Lamarche-Vane N., Li X., Beauchemin N. (2000). Ceacam1-l glycoprotein associates actin cytoskeleton localizes cell-cell contact through activation rho-like gtpases. Mol. Biol. Cell.

[43] Klein T., Proppert S., Sauer M. (2014). Eight years of single-molecule localization microscopy. Histochem. Cell Biol..

[44] Yue S.H., Li P., Guo J.D., Zhou S.G. (2004). Using greedy algorithm: DBSCAN revisited II. J. Zhejiang Univ. Sci..

[45] Lagache T., Lang G., Sauvonnet N., Olivo-Marin J.C. (2013). Analysis of the spatial organization of molecules with robust statistics. PLoS One.

[46] Veatch S.L., Machta B.B., Shelby S.A., Chiang E.N., Holowka D.A., Baird B.A. (2012). Correlation functions quantify super-resolution images and estimate apparent clustering due to over-counting. PLoS One.

[47] Levet F., Hosy E., Kechkar A., Butler C., Beghin A., Choquet D. (2015). SR-tesseler: a method to segment and quantify localization-based super-resolution microscopy data. Nat. Met..

