# Supplementary material for: Correlated STORM-homoFRET imaging reveals highly heterogeneous membrane receptor structures
Source: J Biol Chem. 2022 Sep 5;298(10):102448. doi: 10.1016/j.jbc.2022.102448 (PMC9539790; doi:10.1016/j.jbc.2022.102448)
Supplement: Supplementary information [file mmc1.pdf]

Correlated STORM-homoFRET imaging reveals highly heterogeneous membrane receptor structures

Supplementary information

Method development

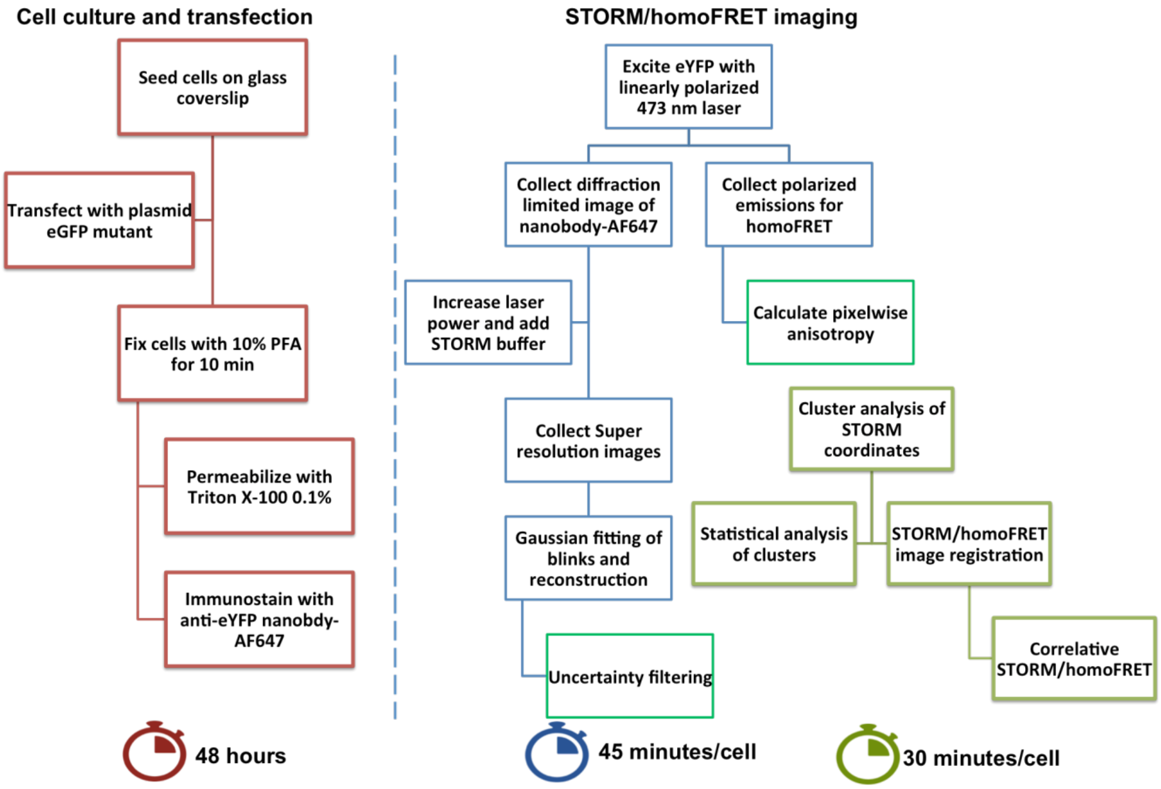

Figure S1. Workflow required to perform STORM/homoFRET measurements in cells transiently transfected with an eYFP fluorescently labeled protein, and immunolabeled using an anti-GFP single domain antibody.

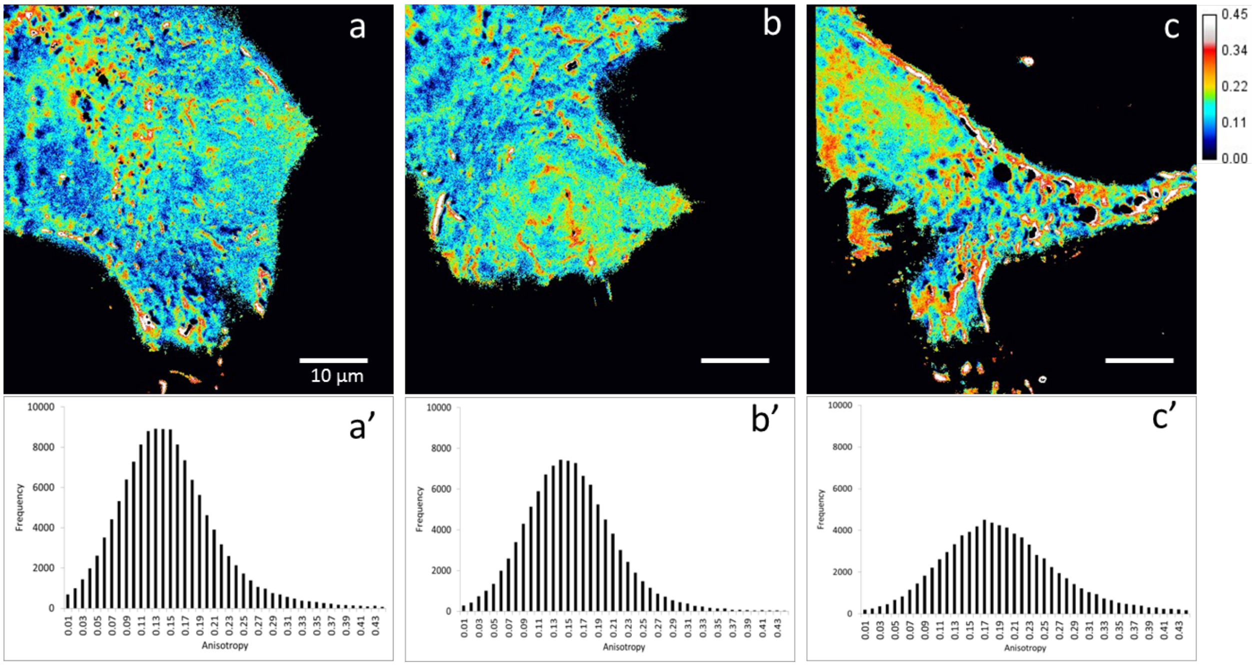

Figure S2. Different cells exhibit slight variation in their distribution of monomers and oligomers. However, the mixture of oligomeric forms remains a common attribute of all CEACAM1-eYFP transfected cells. (**a, b, c)** Anisotropy maps from homoFRET measurements displaying a heterogeneous distribution of CEACAM1 monomers and oligomers**. (a’, b’, c’)** Corresponding anisotropy distributions for each of the associated images.

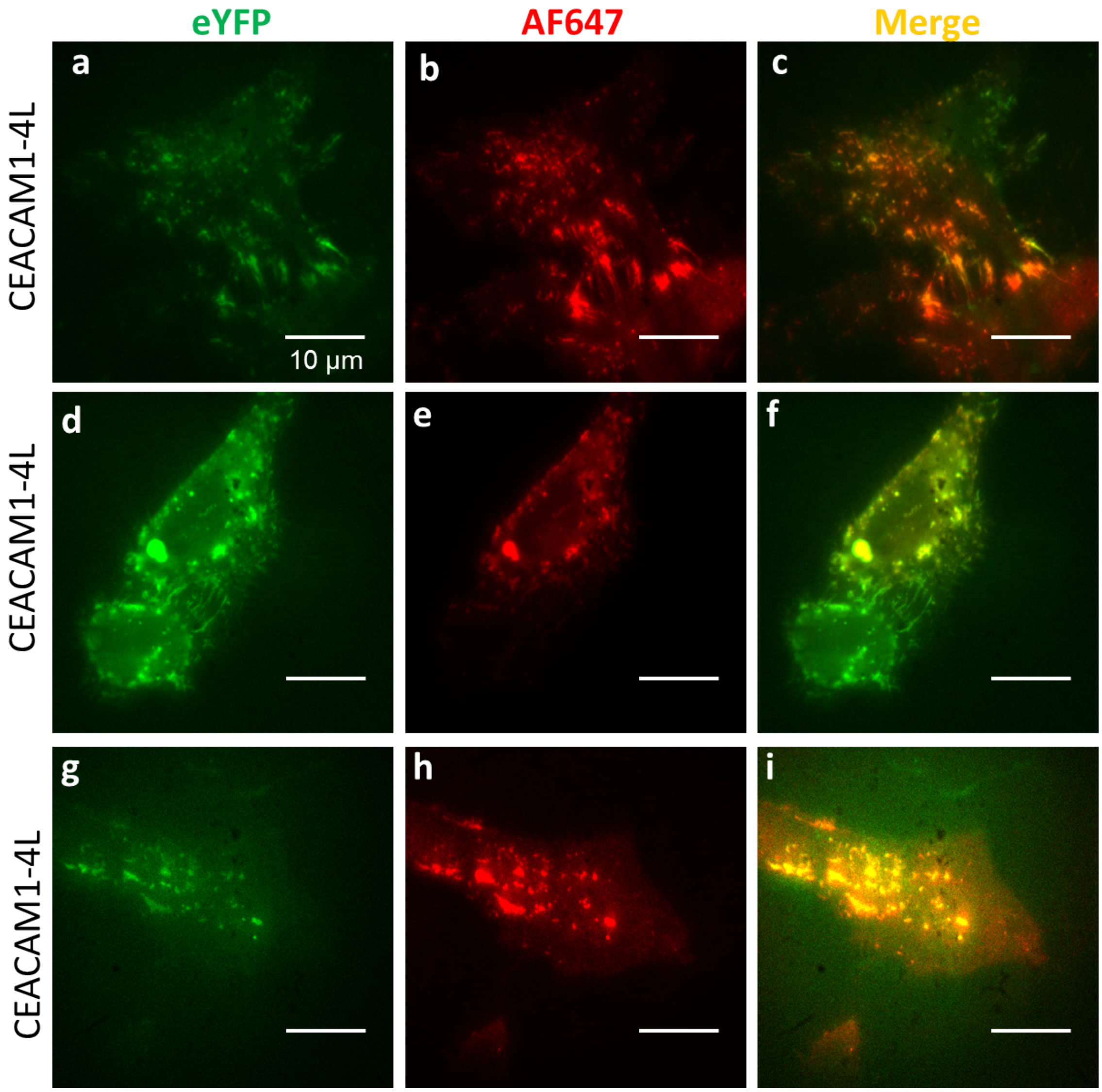

Figure S3. Representative images of the colocalization analysis showing the specificity and accuracy of the single-domain antibody labeling. **(a, d, g)** Transiently transfected HeLa cells with CEACAM1-eYFP, **(b, e, h)** Single domain antibody-AF647 labeled cells,**(c, f,i)** Merged eYFP and AF647 channels display the extent of colocalization. Scale bar: 10 μm

Validation of homoFRET

In order to validate our TIRF-homoFRET measurements, we compared the steady-state anisotropy values for monomeric and tandem-dimeric Venus fluorescent proteins, which are soluble proteins **(Figure S4)**. HeLa cells were transiently transfected with either Venus monomer or Venus tandem-dimer. Cell media was exchanged 18 hours after transfection and the cells were imaged using our TIRF-homoFRET setup.

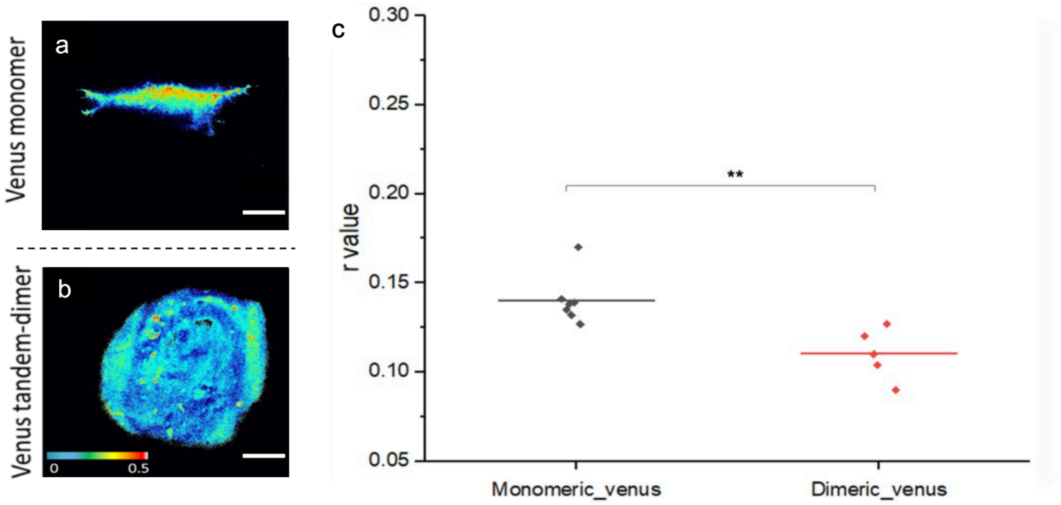

Figure S4. HomoFRET anisotropy control experiment. Representative cells showing the pixel-wise anisotropy map for **(a)** monomeric-Venus and **(b)** tandem-dimer Venus transiently transfected in HeLa cells, **(c)** Average anisotropy plots generated from 7 and 5 cells respectively of the monomeric and tandem-dimeric Venus from 2 replicate experiments. Dots represent individual cells. Assuming a normal distribution, using a two-sample t-Test, we find that $p < 0.005$. Scale bars in panels **a** and **b** denotes 10 µm.

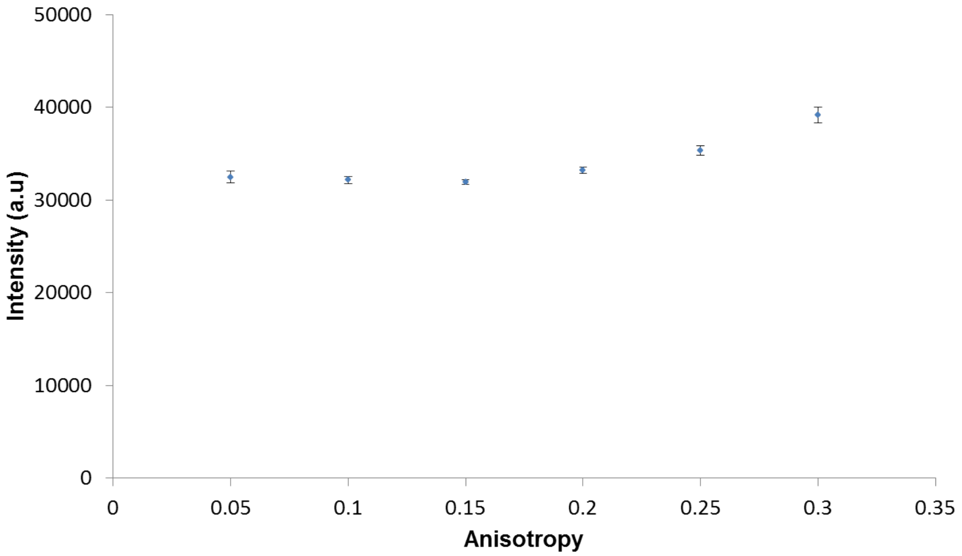

Figure S5. Anisotropy vs Intensity. The relationship between pixel-wise anisotropy and intensity shows that reported anisotropy values are not skewed by intensity but instead represent the ratiometric measurement of anisotropy. Error bars represent standard error of the mean from 6 cells from 2 biological replicates.

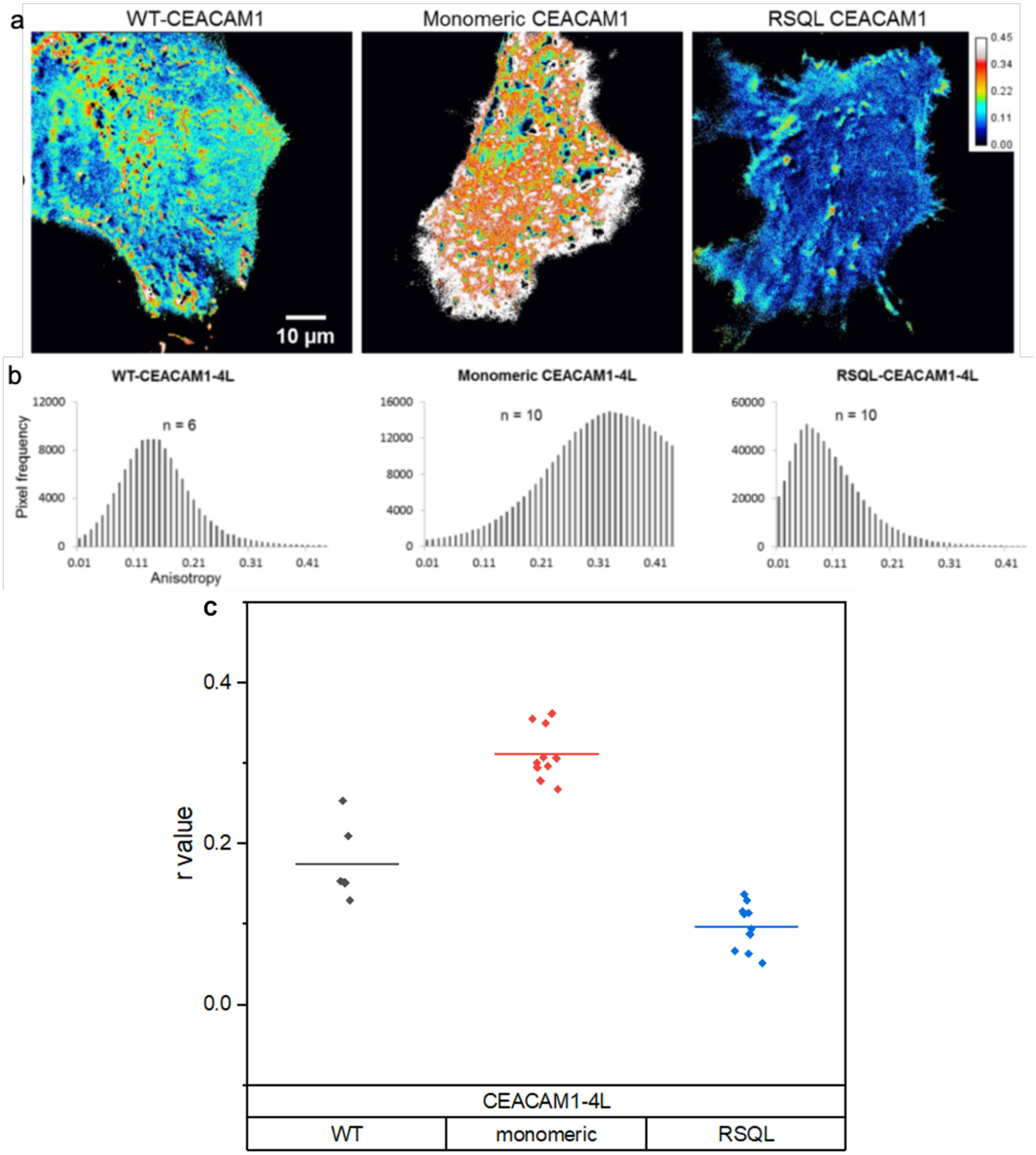

Figure S6. HomoFRET measurements of CEACAM1 WT and mutants shows the validity of the method in capturing expected self-association states. **(a)** Representative images for WT_CEACAM1-4L, monomeric CEACAM1-4L and RSQL-CEACAM1-4L, which is unable to form trans-homophilic interactions, **(b)** Cumulative histograms of intracellular anisotropy distributions for each of the CEACAM1 variants Each n represents an individual cell and the histograms represent the sum of the individual histograms from each cell. (c) Dot-plot displaying the average anisotropy value for each sampled cell per condition. Horizontal lines denote the median anisotropy value for the given condition. Note that image **(a)** and the corresponding histogram

shown in **(b)** are the same as those shown in Figures **S2(a)** and **S2(a')** and are included here as a representative WT-CEACAM1-4L pixel-wise anisotropy data.

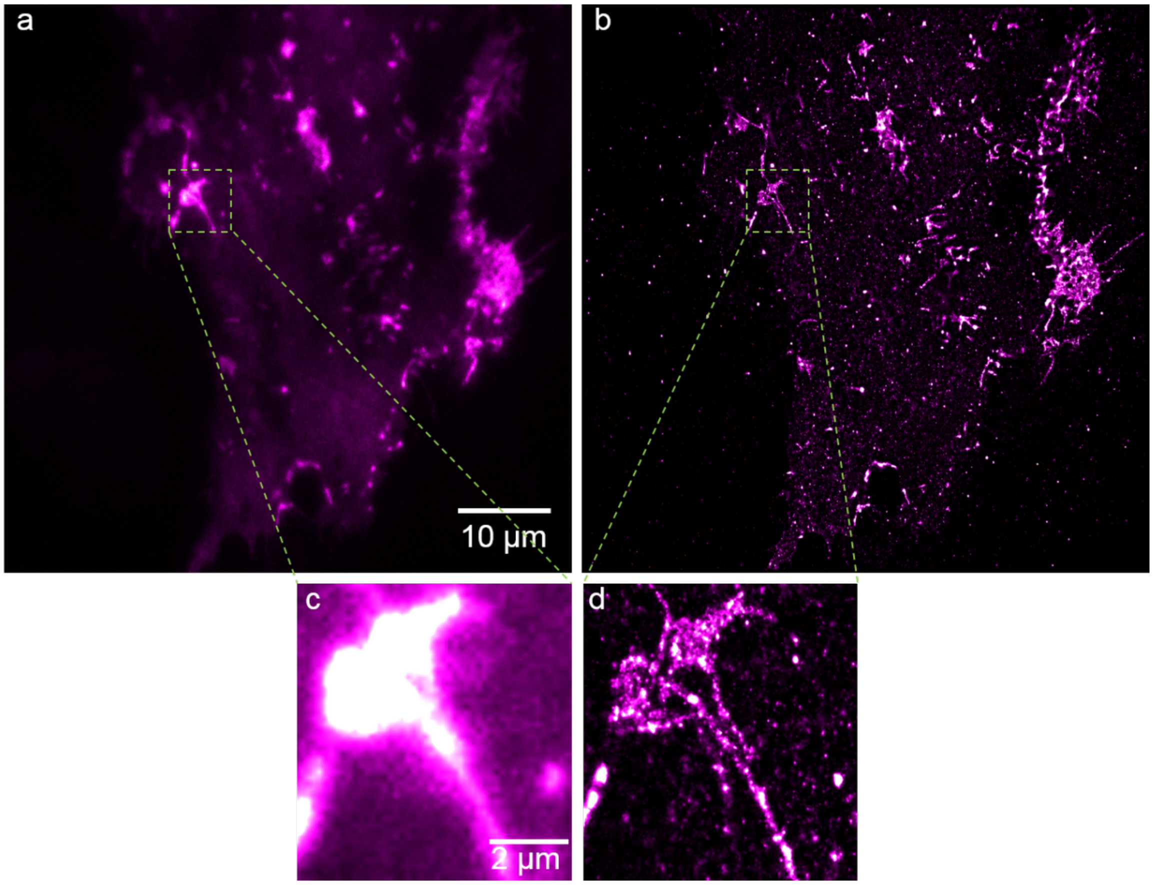

Figure S7. Diffraction-limited and super-resolved CEACAM1-4L
(a) Diffraction-limited image of CEACAM1-4L transiently transfected in HeLa cells, **(b)** Super-resolved image of CEACAM1-4L transiently transfected in HeLa cells, **(c)** Diffraction-limited CEACAM1-4L cluster appears as a homogeneous entitity based on intensity, **(d)** Super-resolved image of CEACAM1-4L cluster reveals a heterogenous protein distribution.

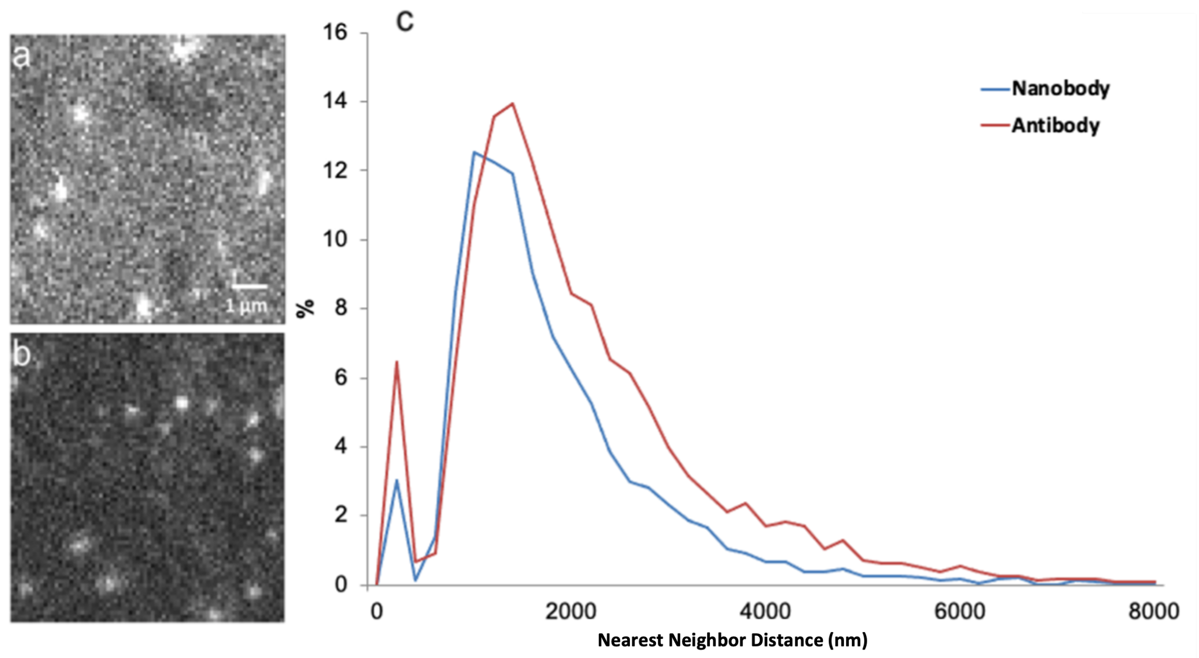

	Nanobody	Antibody
Mean (± s.e.m)	1725 (± 19)	2040 (± 26)

Figure S8. PSF overlap comparison between antibody and nanobody labeling strategies.
(a) Representative image showing blinks generated in a STORM experiment using antibodies (pixel size 127 nm), **(b)** Representative image showing blinks generated using nanobodies, **(c)** Histogram of nearest neighbour distance from blinks in individual frames. 10 frames per stack of 5000 frames taken at 500 frame intervals for each cell with n= 3 cells from independent experiments for each of the labeling strategies. Note that representative images are shown with adjusted contrast so as to display the lower signal to noise ratio in nanobody-labeled images that arises from the lower number of fluorophores per diffraction-limited region.

Since the AF647-labeled nanobody is bound to eYFP, which is itself bound to a flexible linker attached to the cytoplasmic domain of CEACAM1, we expect that there is a small, yet non-negligible fluctuation of the fluorophore's position over the acquisition time. If not accounted for, this might lead to the localization of blinks at slightly different positions for the same fluorophore, which would lead to an overestimation of cluster density. Note that the baseline fluctuation arising from the thermal noise of the EMCCD and laser fluctuation was calculated in order to establish the median instantaneous fluctuation of both eYFP and AF647. Here we characterize the median fluctuation of both eYFP and AF647 in order to establish a lower bound for STORM's localization precision. For this purpose, we captured diffraction-limited images of both eYFP and AF647 with an exposure of 20 ms and a time interval of 30 ms. A Gaussian is fit iteratively to isolated PSFs inside the cell in order to measure the displacement in x and y.

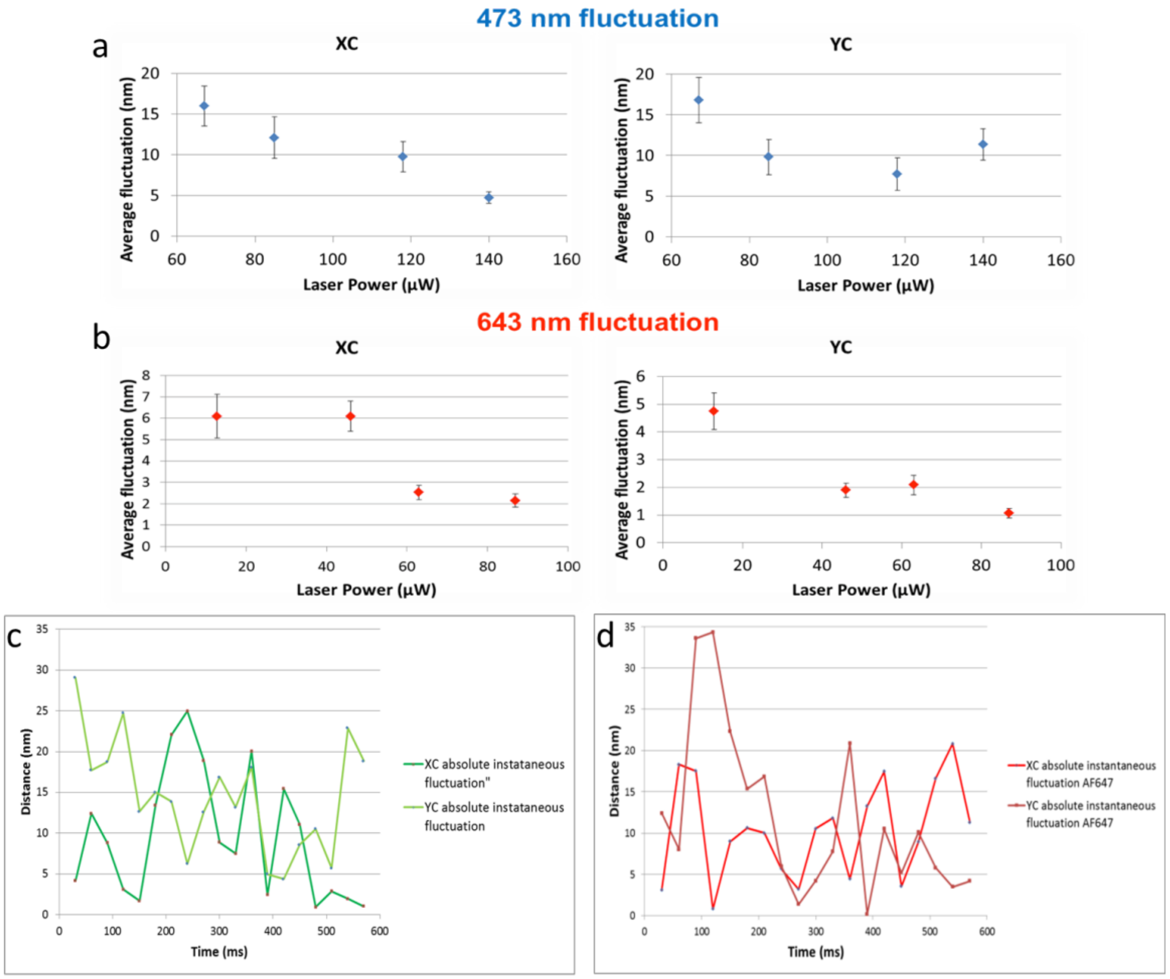

e

	CEACAM1-eYFP		Nanobody-AF647	
	XC	YC	XC	YC
Median Fluctuation (nm)	11	7	9	9

Figure S9. Measure of average **(a)** 473 nm and **(b)** 647 nm fluctuation as a function of laser power. Fluorescent microbeads were imaged in TIRF and the fluctuation was measured by determining the centroid of the bead over time. This experiment shows that intensity fluctuation decreases with higher laser power. **(c)** eYFP absolute instantaneous fluctuation, **(d)** AF647 absolute instantaneous fluctuation, **(e)** Median fluctuation of eYFP and AF647-nanobody conjugates in HeLa cells transiently expressing CEACAM1-4L.

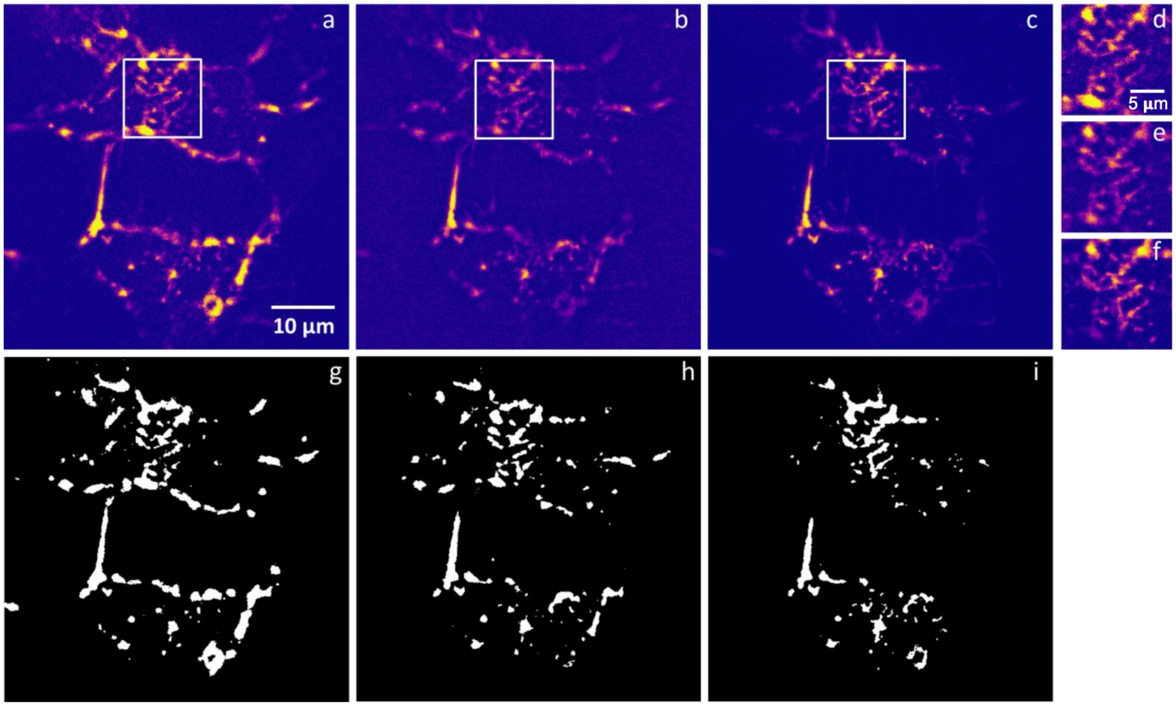

Figure S10. HeLa cells transiently expressing CEACAM1-4L sequentially **(a)** fixed with 4% PFA, **(b)** permeabilized using 0.1% Triton X-100, **(c)** nanobody labeled. Selected ROI for **(d)** fixed, **(e)** permeabilized and **(f)** nanobody-labeled cells. Binary images of **(g)** fixed, **(h)** permeabilized and **(i)** nanobody-labeled cells, respectively. Binary images were obtained by thresholding relative to the fixed-cell image. Certain clusters and regions appear to have disappeared for this reason due to photobleaching. Scale bar shown in **(a)** denotes 10 µm. Images **(a-c, g-i)** are all the same size Scale bar in ROI image **(d)** denotes 5 µm. ROI images **(d-f)** are all the same size

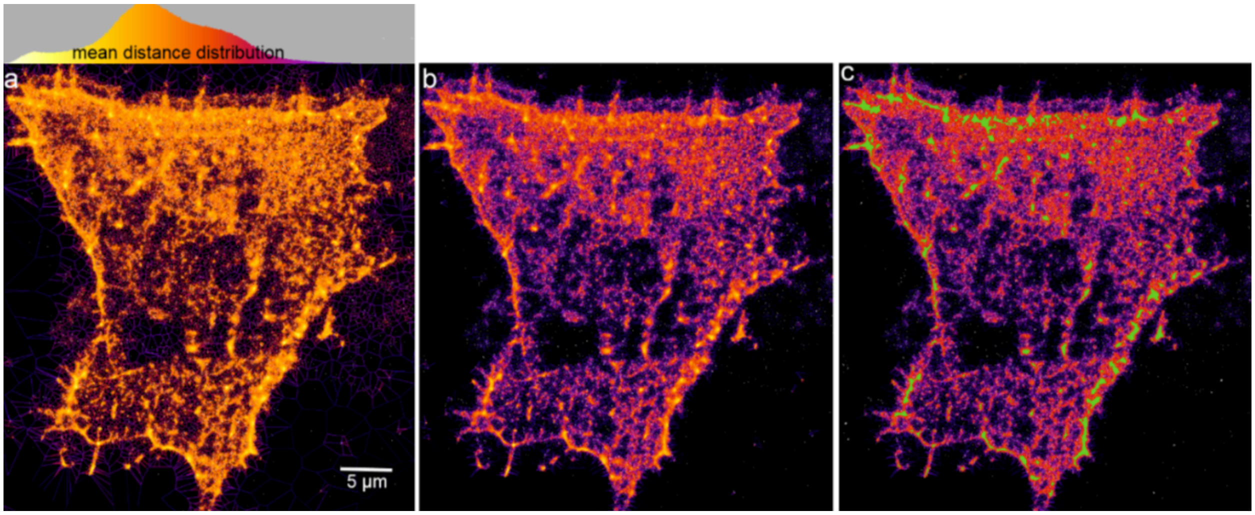

Figure S11. Application of Voronoi tessellation on STORM generated localizations of CEACAM1-4L. **(a)** Voronoi map of all localizations, colour coded for mean distances, (**b)** Voronoi map with background localizations thresholded out, **(c)** Example of clusters (green) that can be segmented using Voronoi tessellation.

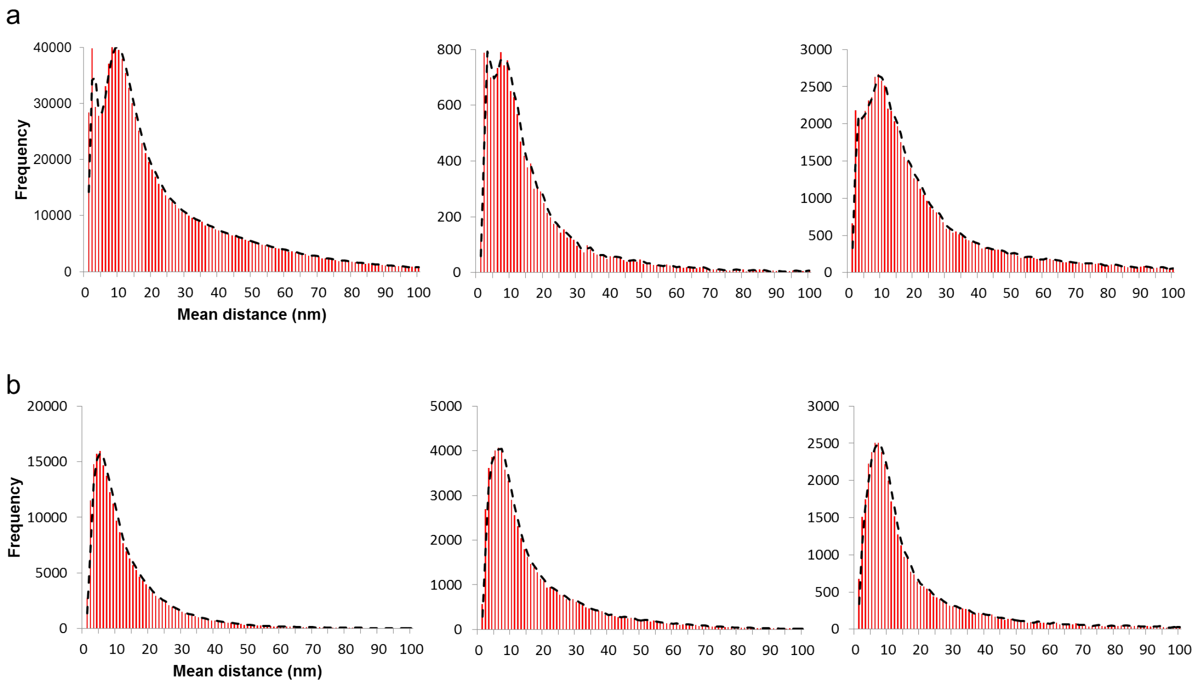

Figure S12. Voronoi tessellation analysis of CEACAM1 highlights that CEACAM1 can arrange its clustering properties depending on the cell's signaling requirements.

(a) Three (3) mean distance distributions from 3 cells from 2 replicate experiments showing that CEACAM1 can cluster into 3 distinct populations, namely: nanoclusters, microclusters and diffuse regions, **(b)** However, in some cells, the nanocluster population is barely resolvable as shown here where only microcluster and diffuse regions can be extracted. Black dotted line represents the moving average (moving average of 2 nm).

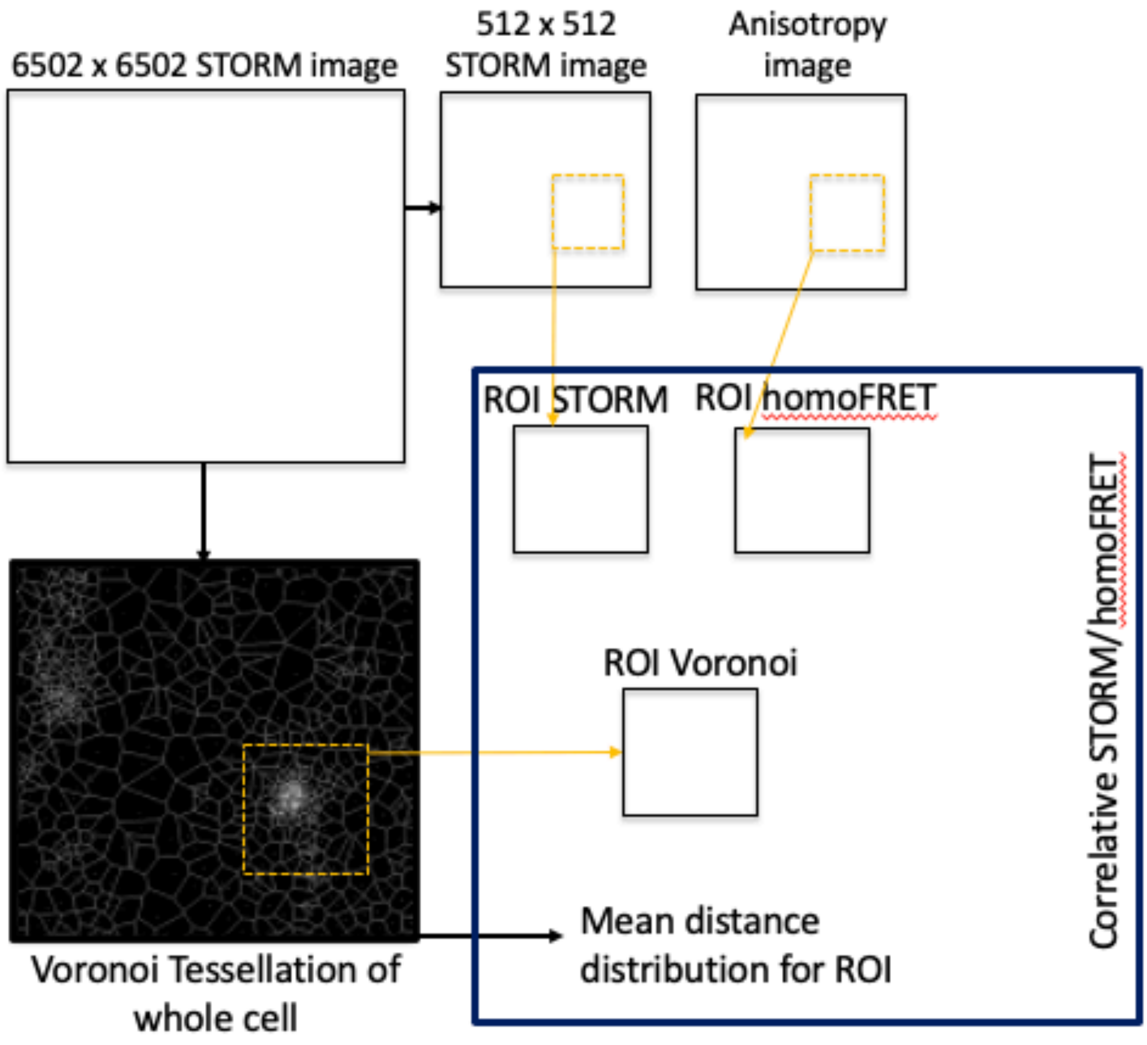

Figure S13. Visual representation of the STORM/homoFRET analysis pipeline. The super-resolved image is rescaled without interpolation into a 512 x 512 image while the coordinates are inputted for cluster analysis using Voronoi Tessellation. ROIs are selected for each the STORM image, anisotropy image and Voronoi segmentation. From these ROIs, we obtain the mean distance statistical distribution and the anisotropy distribution.

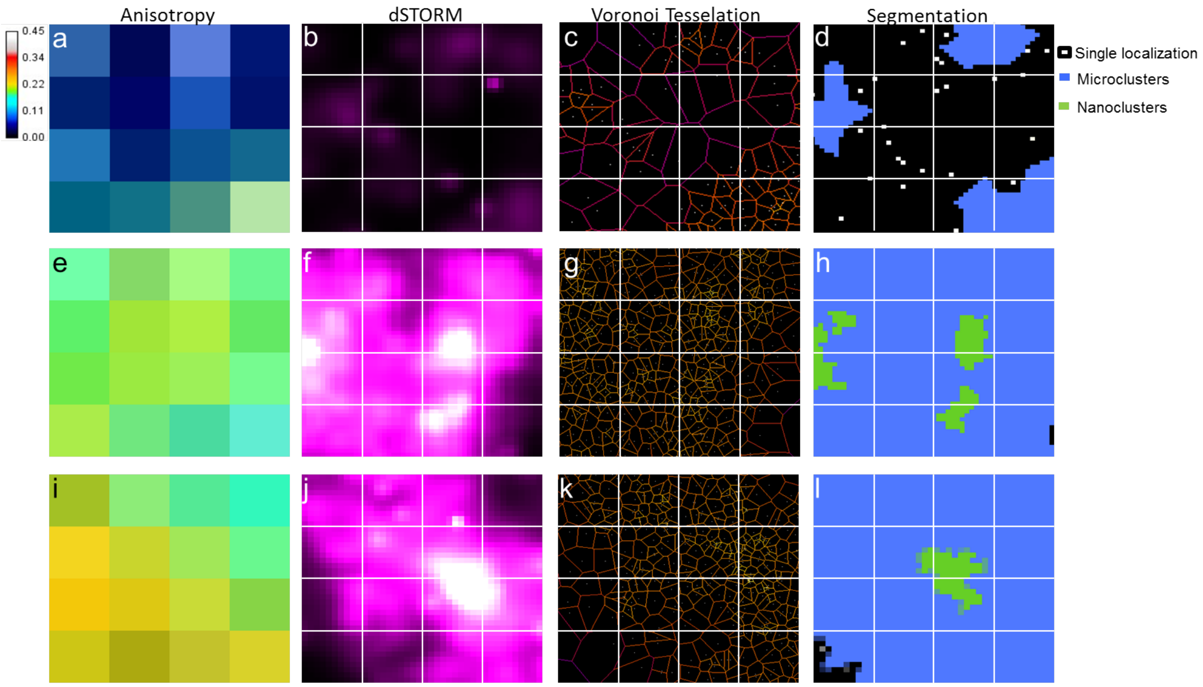

Figure S14. Three distinct ROIs within the same CEACAM1 cluster showing how different anisotropy values correlate with nanoscale spatial distributions obtained via STORM and analyzed through the use of Voronoi tessellation. White lines delimitate diffraction-limited pixel size, with 4 x 4 pixels of 127 nm. **(a)** Anisotropy map CEACAM1-eYFP. **(b)** Super-resolved CEACAM1-AF647. **(c)** Voronoi tessellation clustering. **(d)** Segmentation of the ROI into diffuse and micro-clustered areas. **(e)** Anisotropy map of CEACAM1-eYFP. **(f)** Super-resolved CEACAM1-AF647. **(g)** Voronoi tessellation clustering. **(h)** Segmentation into micro- and nano-clustered areas. **(i)** Anisotropy map of CEACAM1-eYFP. **(j)** Super-resolved CEACAM1-AF647. **(k)** Voronoi tessellation clustering. **(l)** Segmentation into micro- and nano-clustered areas. Note that images shown as (e-h) are the same as those shown in Figure 7 (i-l) and along with the low (a-d) and high (e-h) anisotropy data are meant to illustrate the relationship between pixel-wise anisotropy and spot spatial distribution.

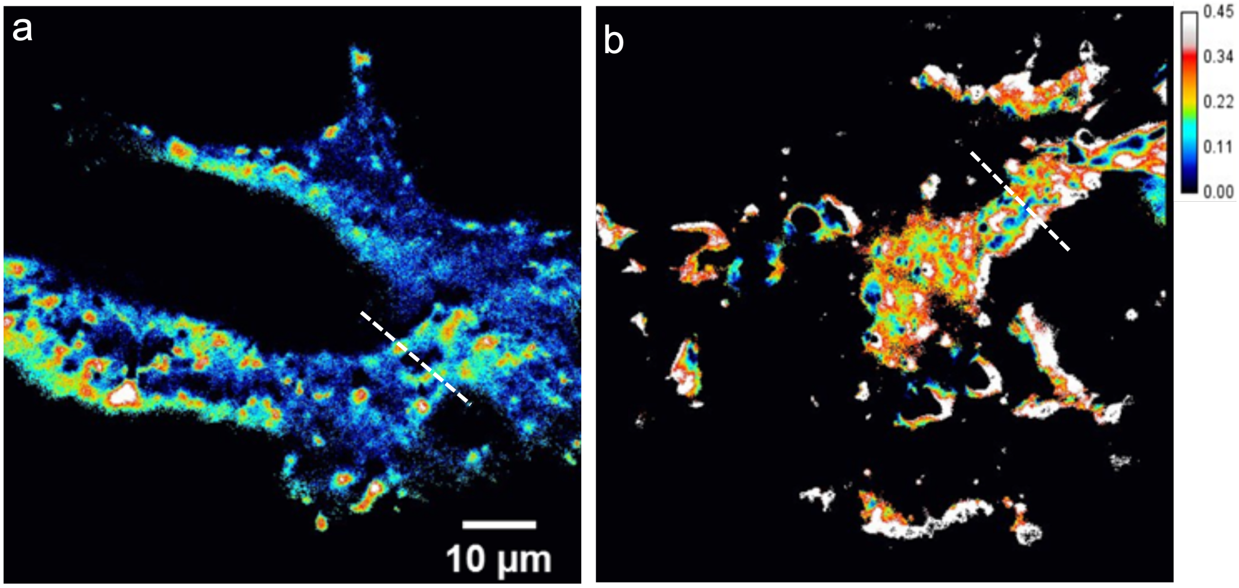

Figure S15. Preliminary work has focused on understanding the self-association and clustering of CEACAM1 at intercellular sites. a) WT-CEACAM1-4L is displaying an enrichment in monomers at intercellular contract sites (broadly delimited by white dotted line), b) monomeric-CEACAM1-4L shows the same enrichment at the intercellular site with the rest of the cell appearing to contain minimal signal. This further indicates a preferential spatial localization of monomers close to intercellular contact sites.

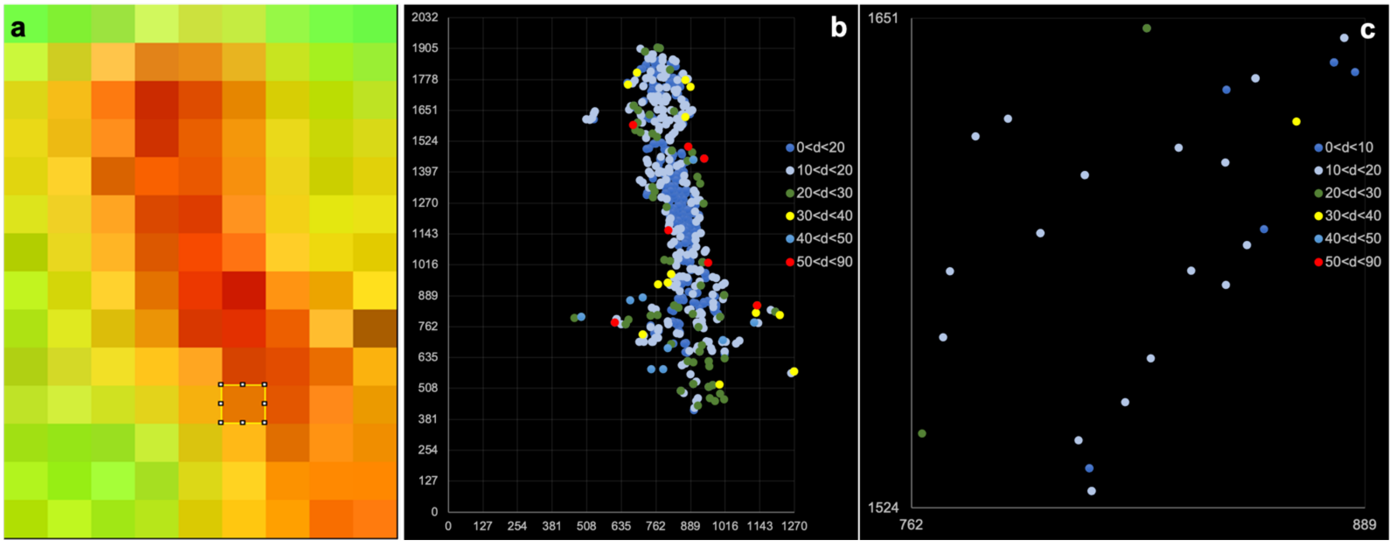

Figure S16. Nearest-neighbour homoFRET/STORM correlation. **(a)** Anisotropy map of a CEACAM1 cluster, **(b)** Color-coded STORM localizations based on NN distances. **(c)** Example ROI (selected box in **(a)** showing individual CEACAM1 localizations (without taking into consideration localization uncertainty).

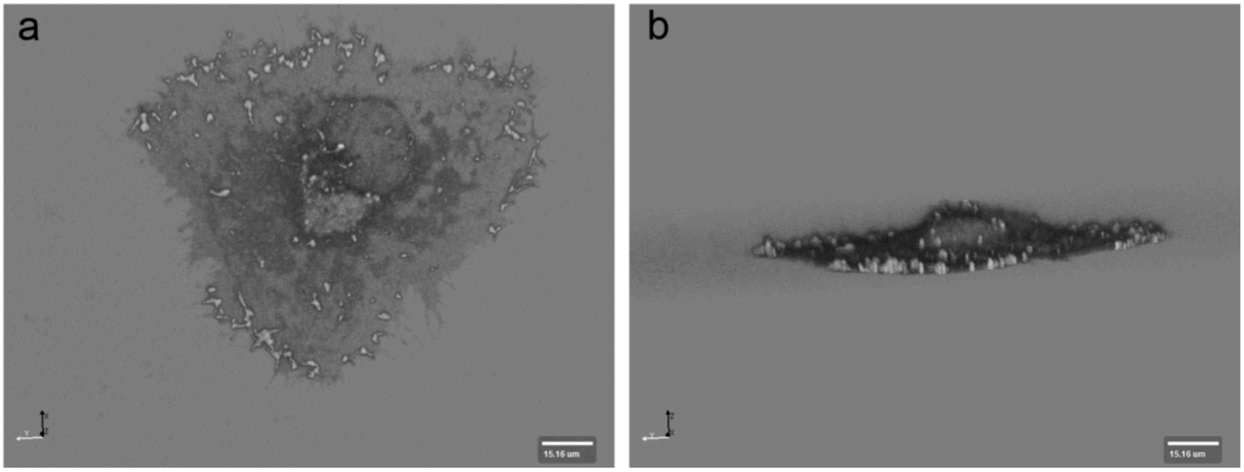

Figure S17: Confocal imaging shows the clear heterogeneity in CEACAM1-4L spatial distribution. **(a)** Top-view of the cell showing presence of CEACAM1-4L around the nucleus and on the apical membrane, **(b)** Side-view of the cell showing that CEACAM1-4L is clearly present in both the apical and basolateral membrane. Scale bar denotes 15.16 µm.

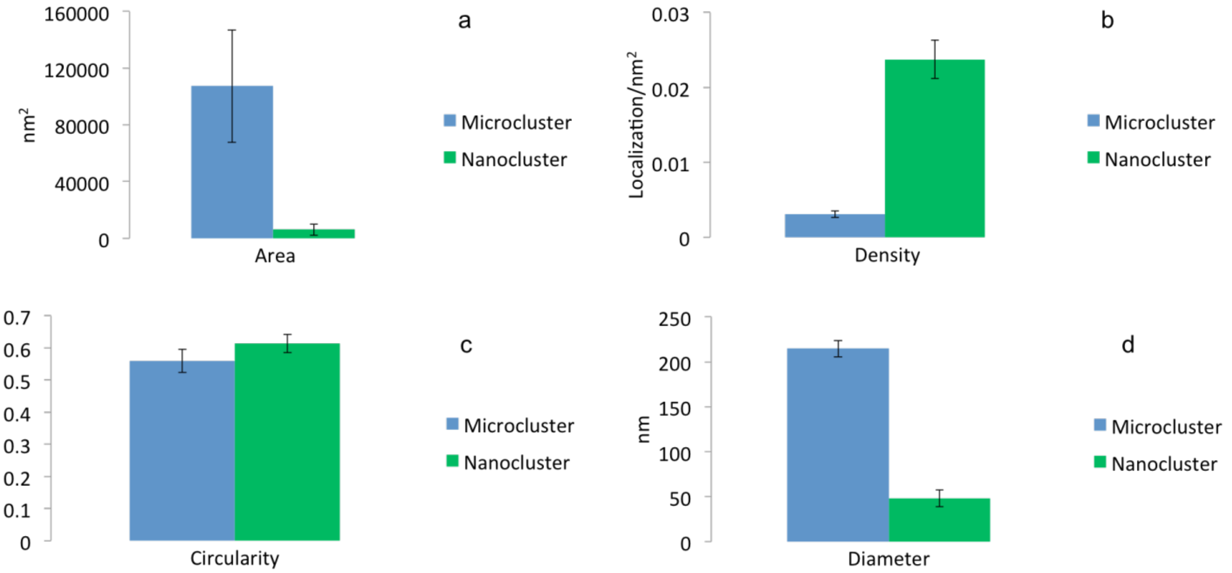

Figure S18. From the various classes of clusters, we can determine their characteristics. Characterization of average cluster area **(a),** density **(b),** circularity **(c)**, and diameter **(d)**, per cluster type, extracted from 8 cells from 3 biological replicates. Error bar represents standard deviation of all clusters per cluster type.
